# Kinetic study of membrane protein interactions: from three to two dimensions

**DOI:** 10.1038/s41598-023-50827-5

**Published:** 2024-01-09

**Authors:** Vladimir Adrien, Myriam Reffay, Nicolas Taulier, Alice Verchère, Laura Monlezun, Martin Picard, Arnaud Ducruix, Isabelle Broutin, Frédéric Pincet, Wladimir Urbach

**Affiliations:** 1 Laboratoire de Physique de l’École normale superieure, École Normale Supérieure, Université Paris Sciences et Lettres, CNRS, Sorbonne Université, Université Paris Cité, F-75005 Paris, France; 2 Department of Infectious Diseases, Avicenne Hospital, AP-HP, Université Sorbonne Paris Nord, Bobigny, France; 3 Université Paris Cité, Inserm UMR-S 1266, Institute of Psychiatry and Neuroscience of Paris (IPNP), Paris, France; 4 Laboratoire Matière et Systèmes Complexes, UMR 7057, CNRS and Université de Paris Cité, 75205 Paris Cedex 13, France; 5 Sorbonne Université, CNRS, INSERM, Laboratoire d’Imagerie Biomédicale-LIB, 75006 Paris, France; 6 Laboratoire CiTCoM, Faculté de Santé, Université Paris Cité, CNRS, 75006 Paris, France; 7 Université Paris Cité, CNRS, Expression Génétique Microbienne, Institut de Biologie Physico-Chimique, Paris, France; 8 Université Paris Cité, Laboratoire de Biologie Physico-Chimique des Protéines Membranaires CNRS UMR7099, 75005 Paris, France; 9 Institut de Biologie Physico-Chimique, Fondation Edmond de Rothschild, 75005 Paris, France

**Keywords:** Biophysical chemistry, Membrane proteins

## Abstract

Molecular interactions are contingent upon the system’s dimensionality. Notably, comprehending the impact of dimensionality on protein–protein interactions holds paramount importance in foreseeing protein behaviour across diverse scenarios, encompassing both solution and membrane environments. Here, we unravel interactions among membrane proteins across various dimensionalities by quantifying their binding rates through fluorescence recovery experiments. Our findings are presented through the examination of two protein systems: streptavidin–biotin and a protein complex constituting a bacterial efflux pump. We present here an original approach for gauging a two-dimensional binding constant between membrane proteins embedded in two opposite membranes. The quotient of protein binding rates in solution and on the membrane represents a metric denoting the exploration distance of the interacting sites—a novel interpretation.

## Introduction

Understanding the strength of protein interactions within disease pathways informs the design of drugs aimed at disrupting these interactions and impeding disease progression. The interactions among proteins are notably influenced by the surrounding space’s availability, which corresponds to its dimensionality. This dependence on dimensionality arises from the system's geometry, which directly shapes the degrees of freedom and thereby influences interaction characteristics.

In lipid bilayers, proteins that interact within the same bilayer (*cis* interactions) or between distinct bilayers (*trans* interactions) can undergo either attractive or repulsive forces according to the charges of neighboring proteins or the surrounding lipid head groups. In three-dimensional systems such as the cytoplasm or extracellular space, interactions among proteins are influenced by a greater number of degrees of freedom compared to bilayers, given their enhanced ability to move and rotate. A scenario of intermediate significance arises when a membrane-bound protein interacts with a soluble counterpart, a common occurrence within cells. Additionally, the system's dimensionality can undergo alteration during protein–protein interactions; for instance, proteins interacting within the cytoplasm might become anchored to the plasma membrane, leading to a shift in the system's geometry and potentially modifying the strength and nature of their interactions^1–5^.

Despite this reality, most prevalent approaches tend to disregard these complexities. They typically examine the interaction of soluble proteins in solution and membrane proteins integrated into supported bilayers or micellar systems designed for soluble proteins^6–8^. Nonetheless, a few techniques permit a direct comparison of interactions involving the same proteins across different dimensionalities^9,10^.

In this work, we present an approach that closely mirrors physiological conditions, utilizing flexible bilayers and employing fluorescence recovery after photobleaching (FRAP)^11^ to quantify the interaction constant, K, between proteins whether embedded within membranes or not.

Our investigation leverages the ${L}_{3}$ (or sponge) phase, conceptualized as a “melted” cubic phase featuring an isotropic structure^12^. This phase comprises a bilayer centered on a minimal (zero mean curvature) surface, dividing space into two interpenetrating solvent labyrinths. Elucidation of this intricate structure derives from diverse scattering data types, encompassing X-rays, neutrons, and light, along with transport properties^13–15^. On a local scale, the ${L}_{3}$ phase’s structure resembles that of the lamellar Lα phase^13^. However, on a larger scale, the infinite bilayer separates two interwoven, equivalent, and self-connected solvent domains^16^. The appearance of the ${L}_{3}$ phase can be theoretically interpreted as a transition from the ordered cubic arrangement of the membrane to a sponge-like membrane structure, as the regular periodic structure of the cubic phase gets perturbed by bilayer thermal fluctuations, introducing defects that drive the transformation into a sponge-like configuration. Spectra obtained from radiation scattering manifests a well-defined Gaussian peak indicating an average characteristic distance, ${d}_{w}$^17^.

The capacity to compare a two-dimensional system when a protein is membrane-anchored and a three-dimensional situation with the protein in solution stands pivotal in comprehensively determining and predicting their physiological conduct. The three scenarios—both proteins in membranes ("2-d"), one protein in a membrane and the other in solution ("2.5-d"), and both proteins in solution ("3-d")—can be comparatively examined within the same protein pair. We demonstrate the full potential of this approach through two examples: (i) the streptavidin/biotin complex, commonly employed as a reference for robust ligand-receptor pairs, and (ii) an efflux pump involved in a resistance mechanism developed by Gram-negative bacteria, specifically *Pseudomonas aeruginosa*^18–20^. In the latter case, we utilize two proteins within the three-protein complex forming the principal efflux pump found in these bacteria: MexA and OprM. MexA^21,22^ functions as a membrane fusion protein (MFP) connected to the inner membrane via a lipid anchor, found in the periplasmic space, while OprM^23^ represents a trimeric outer membrane channel belonging to the outer membrane factor family (OMF). Subsequently, "OprM" denotes the entire trimeric channel. Detailed high-resolution structures have been elucidated for these^23–25^. They interact to form a complex with a two-by-two (2, 4, 6 up to 12) MexA to one OprM^26,27^. More recently, the ternary OprM-MexA-MexB complex was visualized using high-resolution electron microscopy^28^. Interaction kinetics differ across different geometries, contingent upon each protein's degrees of freedom, and the rates of association and dissociation between each geometry are linked by a characteristic length. This characteristic length provides a quantitative measure of the relative ease of protein binding across various types of dimensionality.

## Results

### Method to measure 2-d association

Monitoring in situ the interactions of two membrane-bound molecules is challenging especially when these interactions occur between opposing bilayers. The method we propose here is based on two key considerations: 1. The diffusion coefficient of species depends on their binding state; 2. L_3_ phases allow easy insertion of membrane-bound proteins, maintaining their activity^29^, and calibration of the intermembrane distance^30^. Hence, we propose to obtain the equilibrium constant and kinetic rates between two membrane proteins embedded in bilayers by measuring their diffusion coefficients in a L_3_ phase. One of the two proteins is fluorescently labeled, and we performed Fluorescence Recovery After Fringe Pattern Photobleaching (FRAPP) experiments (see “Materials and methods” for details).

When the bound and unbound states of fluorescent protein are present in the system, the fluorescence intensity recovery displays a double exponential behavior, as long as any temporal changes of the sample are much slower than the time scale of the experiment:

1 $$I\left(t\right)= {I}_{F}\left(1-{e}^{-\frac{t}{{\tau }_{F}}}\right)+{I}_{B}\left(1-{e}^{-\frac{t}{{\tau }_{B}}}\right).$$

The two diffusion times ${\tau }_{F}$ and ${\tau }_{B}$ are respectively attributed to the unbound free protein and the bound complex. The surface concentrations of free and bound protein are extracted from the plateau intensities ${I}_{F}$ and ${I}_{B}$.

We will show below that measuring the diffusion coefficients at various ratios of the two membrane-bound biological objects is sufficient to accurately measure their 2-d dissociation constant, ${K}_{2d}$, on- and off rates (${k}_{on}$ and ${k}_{off}$ respectively) in *trans* and in *cis*.

### 2-d association in trans

#### Diffusion coefficients of free and bound MexA

**Figure 1:**
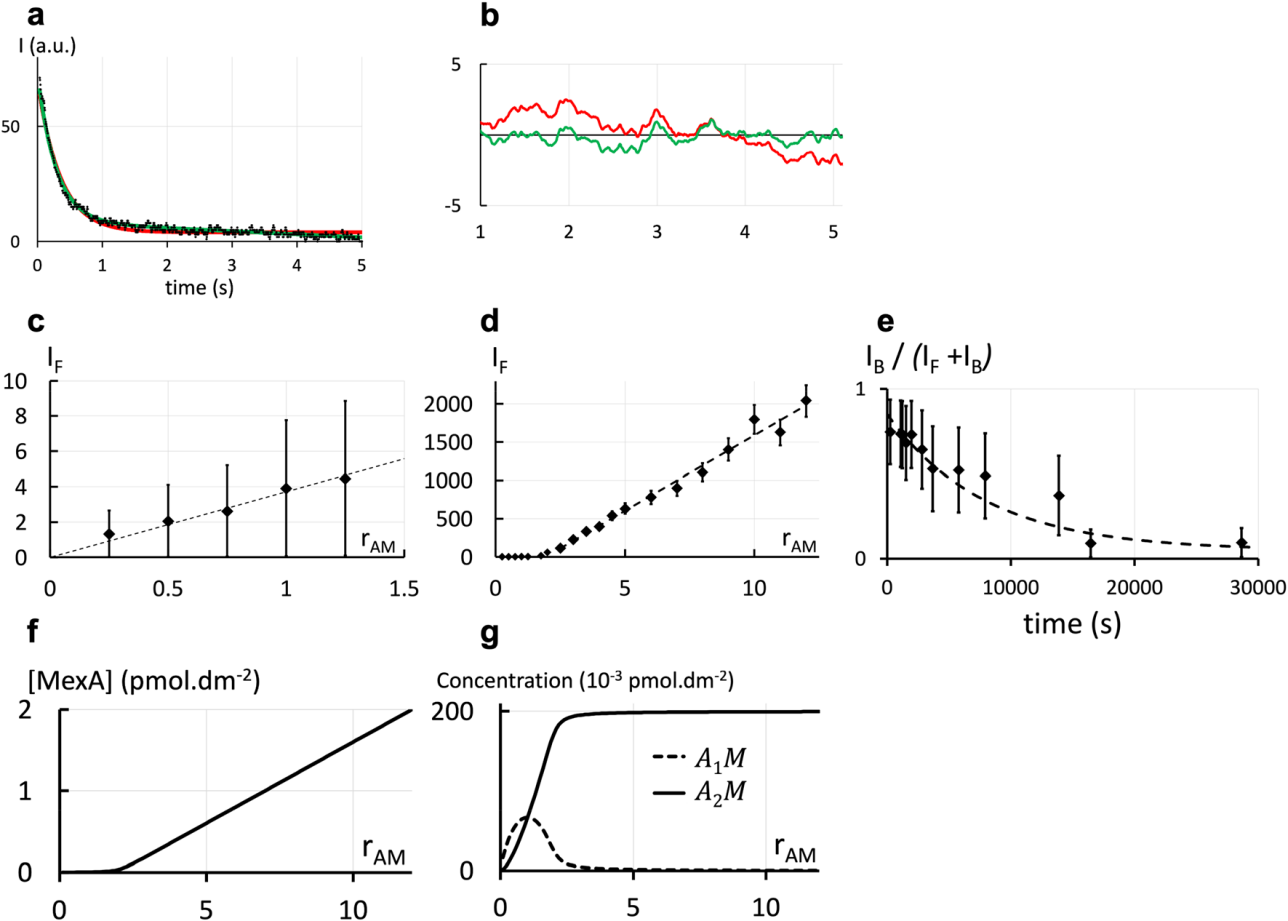
Association between proteins (MexA-OprM) embedded in two opposite bilayers (2-d in *trans*). (**a**) Typical FRAPP fluorescence recovery curve. It tends to zero since our FRAPP device directly subtracts the intensity value in Eq. (1) to the ${I}_{F}+{I}_{B}$ plateau intensity. A bi-exponential fit (green curve) fits better the data than a mono-exponential one (red curve) as shown by the residuals in (**b**), which means that in this case there are two diffusing objects at different mobilities. ${I}_{F}$ and ${I}_{B}$ are the fluorescence intensities respectively of Mex A free and bound to OprM. (**c**) and (**d**) Labelled MexA proteins are introduced in a sample containing OprM. Evolution of ${I}_{F}$ with the initial molar ratio ${r}_{AM}= {n}_{{MexA}_{0}} /{n}_{{OprM}_{0}}$ (where ${n}_{{MexA}_{0}}$ and ${n}_{{OprM}_{0}}$ are the input quantities of MexA and OprM respectively). These results are well-fitted by two different segments (---). The ratio between the two slopes gives a dissociation constant ${K}_{2d}$ = (8.1 ± 0.61) 10^–15^ mol.dm^–2^. (**e**) Temporal evolution of ${I}_{B}/({I}_{F}+{I}_{B}$) when unlabeled MexA proteins are added in large amount to a L_3_ phase containing labelled MexA associated with OprM in stoichiometric condition. The two kinds of MexA proteins are competitors for the interaction with OprM. A fit of the temporal evolution (Eq. (S13) in Supplementary Information, section "2-d association in trans") gives a dissociation rate ${k}_{off}$ = (1.0 ± 0.13) 10^–4^ s^–1^. The deduced association rate is ${k}_{on,2d}$ = (1.23 ± 0.19) 10^10^ mol^–1^.dm^2^.s^–1^. (**f**) Numerical solution of the evolution of the concentration of the free MexA protein with ${r}_{AM}$ (Eq. (S2) in Supplementary Information, Sect. 1, with ${K}_{2d}$ = (8.1 ± 0.61) 10^–15^ mol.dm^–2^). (**g**) Numerical solution of the evolution of the concentration of the bound MexA protein, either in the ${A}_{1}M$ complex (- -) or the ${A}_{2}M$ complex (—) with ${r}_{AM}$ (Eq. (S2) in Supplementary Information, Sect. 1, with ${K}_{2d}$ = (8.1 ± 0.61) 10^–15^ mol.dm^–2^).

In this section, we model the 2-d association of proteins in two opposite bilayers (i.e. in *trans* configuration) with the example of OprM and MexA. To measure the 2-d affinity between OprM and MexA, we inserted them inside a L3 phase with a 20 nm intermembrane distance because we previously found that MexA and OprM interact only when they face each other in opposite membranes separated by 20 ± 2 nm^26^. Since OprM is a large protein complex with transmembrane domains, OprM-bound MexA diffuses much slower than free MexA. Hence, we chose to fluorescently label MexA with FITC. This labeling strategy will ensure that the fluorescence recovery signals can be easily differentiated between free and bound MexA proteins^26,31^. As expected, the recovery signals exhibit a double exponential behavior with two characteristic times for the free and bound states (Fig. 1a and b), that correspond to diffusion coefficients ${D}_{F}$ = 4.5 ± 0.5 µm^2^.s^–1^ and ${D}_{B}$ = 0.6 ± 0.1 µm^2^.s^–1^ respectively. ${D}_{F}$ is the diffusion coefficient previously measured for MexA alone^26^. ${D}_{B}$ is lower than the coefficient of OprM alone (which we measured at 1.3 ± 0.1 µm^2^.s^–1^), probably because bound MexA that is anchored to a bilayer, constraints OprM’s mobility.

To obtain the actual amount of each species, the fluorescence intensities must be converted to proportional concentrations. At pH = 8^26^, bound MexA is found in two types of complexes: one MexA bound to one OprM (${A}_{1}M$) and two MexA bound to one OprM (${A}_{2}M$) that contribute to fluorescence intensity of ${I}_{{A}_{1}M}$ and ${I}_{{A}_{2}M}$ respectively. Because we observed a single characteristic time ${\tau }_{B}$, our FRAPP measurements done at steady-state (~ 3–4 h) did not allow us to distinguish the two proteins complexes ${A}_{1}M$ and ${A}_{2}M$ indicating their diffusion coefficients are similar. This similarity between the two diffusion coefficients was expected since OprM is much larger than MexA and possesses many transmembrane domains. The combined FRAPP of ${A}_{1}M$ and ${A}_{2}M$ is represented by ${I}_{B}$:

2 $${I}_{B}= {I}_{{A}_{1}M}+{I}_{{A}_{2}M} .$$

#### Dissociation constant, on- and off-rates

${K}_{2d}$ Was determined by plotting the variations of ${I}_{F}$ with the initial molar ratio ${r}_{AM}= {n}_{{MexA}_{0}} /{n}_{{OprM}_{0}}$ where ${n}_{{MexA}_{0}}$ and ${n}_{{OprM}_{0}}$ are the input quantities of MexA and OprM respectively (corresponding to concentrations of 0.2 µM and 2 µM). ${I}_{F}$ exhibits a linear behavior before and after ${r}_{AM}$ = 2 (see Fig. 1c and d and Supplementary Information, Sect. 1 for a detailed explanation). The ratio between the two slopes gives ${K}_{2d}$ = 8.1 10^–15^ ± 0.61 10^–15^ mol.dm^–2^.

We then implemented the following protocol to measure the off-rate ${k}_{off}$. OprM and labeled MexA were mixed in the same L_3_ sample in stoichiometric conditions (2 MexA for 1 OprM at 3.2 and 1.6 µM respectively). We prepared the same solution with unlabeled MexA proteins at a concentration 100 times higher. Equal volumes of both solutions were gently mixed. Because of the excess of unlabeled MexA, bound labeled MexA were then replaced by unlabeled one. The kinetics of this replacement process is limited by the unbinding of labeled MexA and thus directly linked to the off-rate of MexA. Recovery curves obtained for different waiting times after mixing directly provided a quantitative measure of this replacement. The variations of ${I}_{B}/{(I}_{F}+{I}_{B})$ are displayed in Fig. 1e. This ratio first seems to be constant and then decreases with time.

A numerical adjustment of the temporal evolution of the ratio ${I}_{B}/{(I}_{F}+{I}_{B})$ (Fig. 1e) gives the off-rate (see Supplementary Information, section "2-d association in trans"): ${k}_{off}$ = (1.0 ± 0.13) 10^–4^ s^–1^. The time resolution of our experiment of the order of 60 s makes it possible to measure an off-rate below 10^–2^ s^–1^ which is comparable with standard techniques like Surface Plasmon Resonance^32^.

The corresponding on-rate ${k}_{on,2d}$ = ${k}_{off}/{K}_{2d}$ is ~ (1.2 ± 0.19) 10^10^ mol^-1^.dm^2^.s^–1^. This is consistent with the fact that with a surface concentration [MexA] ~ 1 pmol.dm^–2^ (see Supplementary Information, section "3-d association") the association time $1/({k}_{on,2d}\left[MexA\right])$ is ~ 80 s. Indeed, our association experiments showed that equilibrium is reached in a time smaller than a few minutes.

### 2-d association in cis

To model 2-d affinities between two proteins embedded in the same membrane (*cis* configuration) we focused on the interactions between a biotinylated transmembrane peptide ${BL}_{12}$ noted $B$, and labeled streptavidin $S$^33,34^. The separation between bilayers is 12 nm which is much larger than the dimension of $S$ thus preventing any *trans* interactions: streptavidin can only be linked to peptides anchored in the same bilayer, as previously demonstrated^26^. $S$ possesses four $B$ binding pockets but can only be bound to a maximum of two $B$ in *cis*-interaction^35^.

In time, the first reaction is the binding of a free soluble $S$ to a membrane-bound $B$ forming a ${B}_{1}S$ complex with an on-rate ${\underline{k}}_{on,2.5d}$. We call this type of reaction with a soluble protein binding a membrane-anchored “2.5-d reactions”. This will be discussed in the next section. The second reaction is the association between two peptides diffusing in the same membrane, a ${B}_{1}S$ to a free $B$ forming a ${B}_{2}S$ complex with an on-rate ${\underline{k}}_{on,2d}$. The corresponding dissociation reactions are not considered, as the lifetimes $\tau$ of the complexes between $S$ and $B$ ($\tau$ ≈ 10^7^ s)^36^ are much longer than the timescale of the experiment (~ 3–4 h). We assume ${\underline{k}}_{off}\approx 0$, thus the equilibrium is reached when $\left[B\right]=0$.

FRAPP measurements on FITC-labelled $S$ allow distinguishing between the free $S$, the complexes ${B}_{1}S$ and ${B}_{2}S$ as their diffusion coefficients are ${D}_{0S}$ = 50 ± 2.4 µm^2^.s^–1^, ${D}_{1S}$ = 3.2 ± 0.2 µm^2^.s^–1^ and ${D}_{2S}$ = 1.6 ± 0.1 µm^2^.s^–1^respectively. The corresponding plateau intensities from the recovery signal are called ${I}_{0S}$, ${I}_{1S}$ and ${I}_{2S}$. We checked that [${B}_{1}S$] and [${B}_{2}S$] are proportional to ${I}_{1S}$ and ${I}_{2S}$ (see Supplementary Information, Sect. 4).

**Figure 2:**
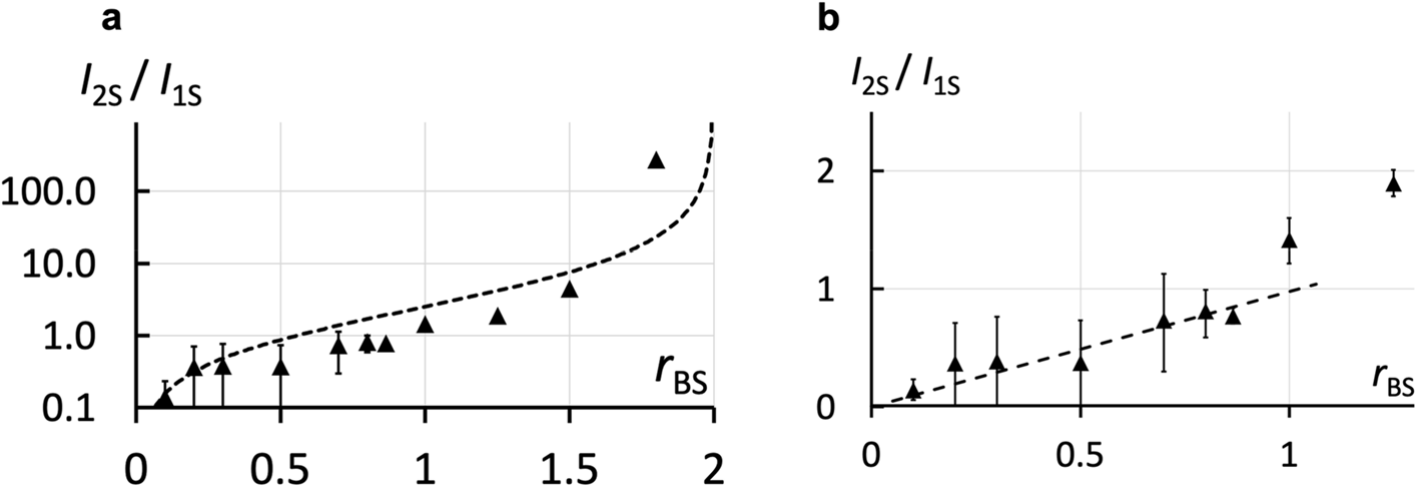
Association between proteins (Streptavidin–biotin) anchored in the same bilayer (2-d in *cis*). ${I}_{1S}$ and ${I}_{2S}$ are the fluorescence intensities respectively of an $S$ bound to a membrane-anchored $B$ forming a ${B}_{1}S$ complex and a ${B}_{1}S$ to a second membrane-anchored $B$ forming a ${B}_{2}S$ complex. (**a**) Results obtained for the variation of the ratio between the intensities ${I}_{2S}/{I}_{1S}$ for different initial molar ratios ${r}_{BS}={n}_{{B}_{0}}/{n}_{{S}_{0}}$ (where ${n}_{{B}_{0}}$ and ${n}_{{S}_{0}}$ are the initial quantities of $B$ and $S$ respectively). Results are compared over a wider range of ${r}_{BS}$ to numerical models from kinetic equations (see Eq. (S23) in Supplementary Information, Sect. 5) performed for ${\underline{k}}_{on,2.5d}$= 10^6^ M^–1^.s^–1^ and ${\underline{k}}_{on,2d}$ = 2.5.10^13^ mol^–1^.dm^2^.s^–1^ (- -) Error bars are not visible when they are lower than the size of the dot. (**b**) For small ${r}_{BS}$ values, ${I}_{2S}/{I}_{1S}$ varies linearly with ${r}_{BS}$ with a slope proportional to ${\underline{k}}_{on,2.5d}/{\underline{k}}_{on,2d}$ and a straight line adjustment (- -) gives ${\underline{k}}_{on,2.5d}/{\underline{k}}_{on,2d}$ = 6.4 ± 0.5 nm.

We introduced a constant concentration of streptavidin ${S}_{0}$ = 9.7 10^–7^ M and varied the concentration of $B$. Figure 2a shows the evolution of ${I}_{2S}/{I}_{1S}$ versus the initial molar ratio ${r}_{BS}={n}_{{B}_{0}}/{n}_{{S}_{0}}$ where ${n}_{{B}_{0}}$ and ${n}_{{S}_{0}}$ are the initial quantities of $B$ and $S$ respectively. This ratio is increased by varying the peptide quantity. At small ${r}_{BS}$ (large excess of $S$), the concentration of $S$ is almost constant and equal to the initial concentration ${S}_{0}$, and we can consider that its bound fraction is negligible. We thus predict (see Supplementary Information, Sect. 5) that ${I}_{2S}/{I}_{1S}$ should vary linearly with a slope proportional to ${\underline{k}}_{on,2d}/{\underline{k}}_{on,2.5d}.$ The data are indeed linear (see Fig. 2b) and lead to ${\underline{k}}_{on,2.5d}/{\underline{k}}_{on,2d}$ = 6.4 ± 0.5 nm. Using the known value of ${\underline{k}}_{on,2.5d}$ (~ 10^6^–1.3.10^7^ M^-1^.s^–1^)^37,38^, we find that ${\underline{k}}_{on,2d}$~1–2.10^14^ mol^–1^.dm^2^.s^–1^. Finally, we plotted the predicted variations (see Supplementary Information, Sect. 6) of ${I}_{2S}/{I}_{1S}$ over a wider range of ${r}_{BS}$ using ${\underline{k}}_{on,2.5d}/{\underline{k}}_{on,2d}$ measured for small ${r}_{BS}$. The resulting curves nicely fit the experimental data, confirming the validity of our approach (Fig. 2).

### 2.5-d association

Here we study the interactions between a membrane bound protein and a partner freely diffusing in the intermembrane space. As mentioned in the previous section, this kind of “2.5-d” association for the $S$/$B$ pair has already been measured with different techniques^37,38^. Hence, we will focus on OprM and MexA using an FITC-labeled soluble mutant of MexA (mMexA) which has a deleted unique N-terminal cysteine preventing anchorage to the membrane. mMexA is solubilized in the intermembrane space of the L_3_ phase^39^ in which an OprM is embedded. In this situation, MexA has more degrees of freedom in comparison to when it is embedded in the membrane.

In a first experiment, we tested mMexA diffusion alone (at 0.2 µM) in the L_3_ phase and measured a single diffusion coefficient, $D$ **=** 20 ± 1 µm^2^.s^–1^ which is consistent with the diffusion of a soluble element in a L_3_ phase^40^. In another experiment, a large excess of OprM protein was added to the L_3_ phase at 2 µM with an initial molar ratio ${r}_{mAM}=$ ${n}_{{mMexA}_{0}}/{n}_{{OprM}_{0}}$ = 0.1, where ${n}_{{mMexA}_{0}}$ and ${n}_{{OprM}_{0}}$ are the input concentrations of mMexA and OprM). As in 2-d experiment, the recovery signals exhibit a double exponential behavior (Eq. 1).

The two diffusion times ${\tau }_{F}$ and ${\tau }_{B}$ are related to diffusion coefficient values of ${D}_{F}$ = 20 µm^2^.s^–1^ and ${D}_{B}$ = 1.4 µm^2^.s^–1^. ${D}_{F}$ is the diffusion coefficient of mMexA diffusing “freely” in the intermembrane space. ${D}_{B}$ corresponds exactly to the diffusion coefficient of OprM alone and we associate this value to mMexA bounded to OprM. Since the OprM protein is in large excess (${r}_{mAM}$ = 0.1, we assume here that only one mMexA protein can bind an OprM. This assumption is supported by the fact that in 2-d geometry, when ${r}_{AM}$ < 0,5, the ${A}_{1}M$ complex was dominant (Fig. 1c).

**Figure 3:**
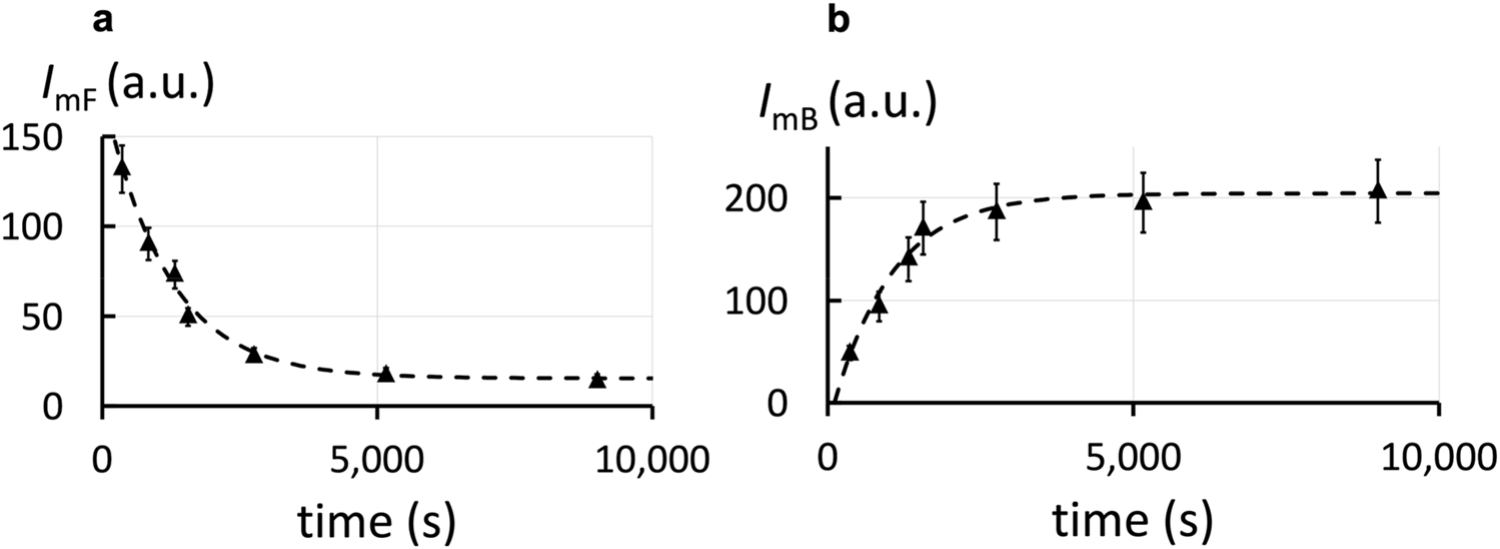
Association between proteins (mMexA-OprM) when MexA is soluble and OprM is embedded in the bilayer (2.5-d). (**a**) Temporal evolution of the intensity ${I}_{mF}$ of free mMexA. Results are fitted by an exponential law (Eq. (S42) in Supplementary Information, Sect. 7). It provides the characteristic time $\tau$ of 1150 ± 119 s. (**b**) Evolution of the intensity ${I}_{mB}$ of mMexA bound to OprM with time. Results are fitted by an exponential law (Eq. S42). It provides the characteristic time $\tau$ of 973 ± 151 s. These two results are compatible. The mean time $\tau$ is deduced: $\tau$ = 1061 ± 136 s.

The measured intensities ${I}_{mF}$ and ${I}_{mB}$ corresponding to each diffusion time are proportional to the number of the free and bound mMexA. We obtained FRAPP signals for different waiting times after the initial mixing of the components. The intensities ${I}_{mF}$ and ${I}_{mB}$ are changing with time: ${I}_{mF}$ decreases while ${I}_{mB}$ increases (Fig. 3a and b) attesting the formation of the $m{A}_{1}M$ complex.

The kinetics of the reaction can be quantitatively described (see Supplementary Information, Sect. 7). The on-rate, ${k}_{on,2.5d}$, and off-rate, ${k}_{off}$, can be obtained by a simultaneous fit of ${I}_{mF}/{I}_{mB}$ and ${{I}_{mF}+I}_{mB}$ over time. The deduced association and dissociation rates are ${k}_{on,2.5d}$ = 131 ± 17 M^–1^.s^–1^ and ${k}_{off}$ = (0.6 ± 0.2) 10^–4^ s^-1^. The small ${k}_{on,2.5d}$ value, compared to common receptor-ligand associations (on-rates of the order of 10^5^–10^7^ M^–1^.s^–1^)^41^, shows that the mMexA-OprM association in the 2.5-d geometry has a low probability to occur. Still, the value of ${k}_{off}$ corresponds to common receptor-ligand associations^41^ and gives a lifetime of the bond of about 4.6 h indicating the mMexA-OprM complex is highly stable with a dissociation energy barrier of approximately 30 ${k}_{B}T$^42^. Furthermore, this ${k}_{off}$ value is compatible with the one found in the 2-d geometry and shows that the off-rate does not depend on the geometry. Finally, we can deduce the dissociation constant: ${{K}_{2.5d}=k}_{off}/{k}_{on,2.5d}$ = (4.6 ± 1.5)0.10^–7^ M. It corresponds to a relatively low affinity for membrane proteins associations. This range is comparable to the ones obtained for interactions between acid sialic and membrane receptors^43^, dimerization of the glycophorinA^44^ or various enzyme and substrate interactions^41^.

### 3-d association

**Figure 4:**
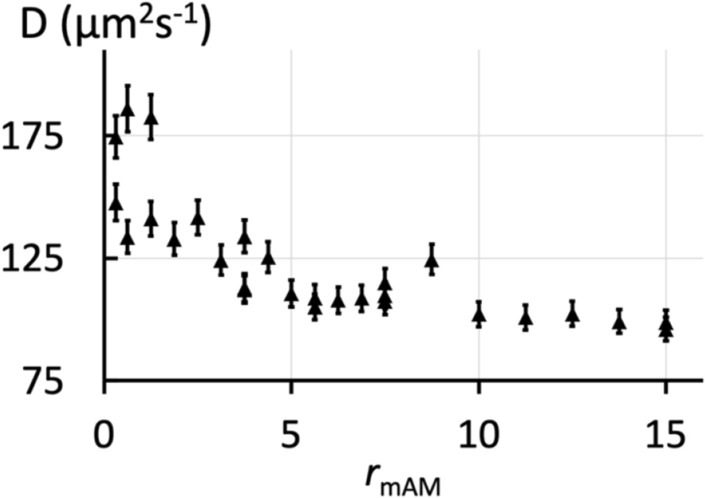
Association between proteins (mMexA-OprM) solubilized in solution (3-d configuration). Variation of diffusion coefficients $D$ with ${r}_{mAM}={n}_{{mMexA}_{0}} /{n}_{{OprM}_{0}}$ (where ${n}_{m{MexA}_{0}}$ and ${n}_{{OprM}_{0}}$ are the input quantities of mMexA and OprM respectively) of FITC-labeled mMexA in solution in the presence of OprM at a concentration of 0.8 μM after 24 h of mixing both proteins. The high values of the diffusion coefficients indicate that mMexA does not interact with OprM. Their decrease with concentration is attributed to possible dimerization or limited aggregation of mMexA.

We used the standard micellar approach to study the interaction between mMexA and OprM in the same saline buffer used to prepare the L_3_ phase. OprM was solubilized in β-OG at 0.9% (w/vol). The concentration of OprM was kept constant (0.8 μM) while the mMexA rate increased (${r}_{mAM}$ from 0 to 15). After 24 h, for all the values of ${r}_{mAM}$, we observed single exponential fluorescence recovery curves, corresponding to diffusion coefficients, $D$, equal to the ones measured when mMexA is alone in solution at the same concentrations (Fig. 4). According to Stokes–Einstein equation, the order of magnitude of the diffusion coefficients correspond to a radius of 2.3 ± 0.1 nm for mMexA which is consistent with its crystal structure^21^. If MexA were linked to OprM, this would slow down the diffusion of MexA. We would then observe a double exponential corresponding to two diffusion coefficients, the free and bound mMexA. One of the exponential due to the free mMexA would represent a diffusion coefficient of $D$ equal to the diffusion coefficients of mMexA alone in solution at the same concentrations. Because of the large size of OprM compared to the micelle radius, the second exponential, due to bound mMexA, would represent a diffusion coefficient similar to that of OprM alone at the same concentration, i.e. $D$ = 32 ± 0.4 μm2.s^–1^, which corresponds to a Stokes radius of 6.7 nm consistent with the crystal structure of OprM^23,45^. Hence, our results suggest that mMexA does not interact with OprM in 3-d geometry. This was confirmed by blue native polyacrylamide gel electrophoresis^27^. The value of ${K}_{3d}$ is therefore must be much higher than ${K}_{2.5d}$, indicating a lower affinity in three dimensions.

## Discussion

### 2-d association in trans

We presented in this article a simple and original approach to measure the affinity between two membrane proteins in the *trans* configuration. Other methods can be mechanical (e.g. micromanipulation assays between two cells^9,10^. Our approach allows us to measure 2-d affinities up to 10^–14^ mol.dm^–2^. The tunable inter-bilayer distance from 5 to 30 nm of the L_3_ phase makes it possible to study other membrane protein *trans* interactions in gram-negative bacteria (both membranes being separated by 20 nm) or mitochondria (22 nm), trans-synaptic complexes (the height of a chemical synaptic cleft being of 10–30 nm)^46–49^, lipid transfer proteins and other complexes that connect the endoplasmic reticulum to plasma membrane (again with a gap typically within 10 to 30 nm)^50,51^ or the two membranes of the autophagosome (10–30 nm also)^52^.

We solved the equilibrium (Eq. (S1) in Supplementary Information) with ${K}_{2d}$ = (8.1 ± 0.61) 10^–15^ mol.dm^-2^ (Fig. 1f and g). For ${r}_{AM}$ > 2, only the ${A}_{2}M$ complex is present; again, this confirms our previous assumption: we considered [${A}_{1}M$] to be negligible compared to [${A}_{2}M$].

Furthermore, Fig. 1g explains why no significant break in the slope of ${I}_{F}$ was observed in Fig. 1c when ${r}_{AM}$ = 1. It might have been supposed that the slope when ${r}_{AM}$ < 1 would be the same as when ${r}_{AM}$ > 2, if MexA proteins would only form ${A}_{1}M$ complexes. This is not the case because when ${r}_{AM}$ < 1, ${A}_{2}M$ cannot be neglected. Probable quenching effects even at low ${r}_{AM}$ explain why the slope is lower also for ${r}_{AM}$ < 1 than for ${r}_{AM}$ > 2. Our results also allow us to predict that all ${A}_{2}M$ complexes are assembled in the bacteria. Narita et al. count 2500 MexA and 1200 OprM per bacteria. Modeling a bacteria as a sphere of radius 1 µm, the surface concentration in MexA and OprM is ~ 10^–12^ mol.dm^–2^^53^. The 2-d dissociation constant measured here, ${K}_{2d}$ = (8.1 ± 0.61) 10^–15^ mol.dm^–2^, indicate that all MexA and OprM will associate in the bacteria if all OprM proteins are accessible.

### Significance of the characteristic length linking 2-d to 2.5-d Association

**Figure 5:**
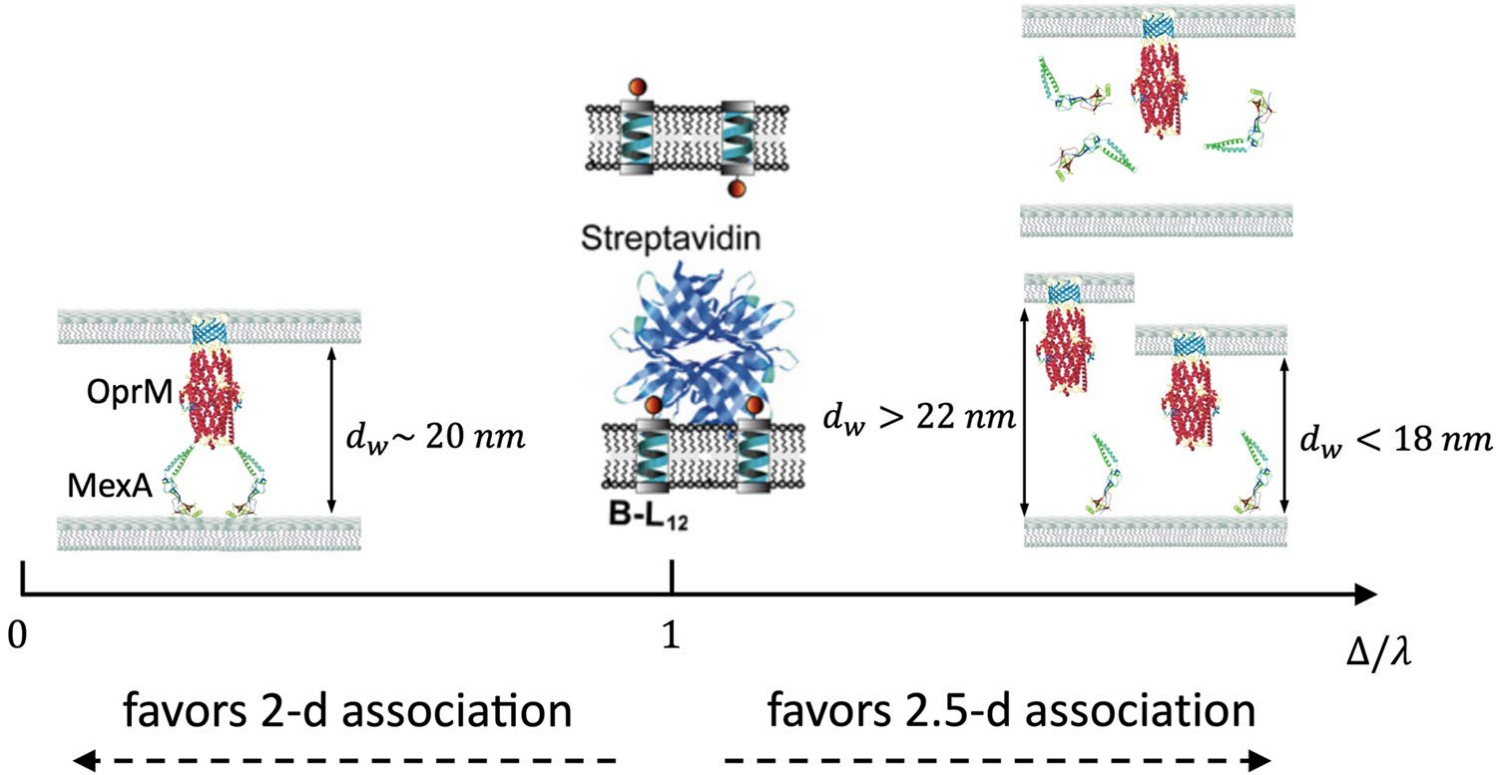
Graphical representation of different geometries of protein interaction with $\Delta /\lambda$. The ratio > 1, it favors 2.5-d association, whereas the ratio < 1, favors 2-d interaction.

The ratio between the “2.5-d” and the 2-d association rates provides a characteristic length ${\Delta =k}_{on,2.5d}/{k}_{on,2d}$. It reflects how much the binding of both proteins to membranes facilitate their association: the smaller $\Delta$, the most favorable the 2-d interactions compared to the 2.5-d interactions. More precisely, in 2.5-d, one of the proteins freely diffuses in the bulk while in 2-d, it remains anchored to the membrane. Hence, the apparent volume concentration of the protein in the 2-d geometry is about: $\left[P\right]=\left\{P\right\}/\lambda$ where $\left\{P\right\}$ is the surface concentration (in mol.dm^-2^) and $\lambda$ is the typical distance between the anchoring point and the binding site, which, in general, is commensurate with the protein size. The 2-d on-rate deduced from this apparent volume concentration and the 2.5-d on-rate would then be: ${{k}_{on,2d apparent} =k}_{on,2.5d}/\lambda$. Hence, if $\Delta <\lambda$, the pair will more easily associate in 2-d while if $\Delta >\lambda$ the two partners will better bind in 2.5-d (Fig. 5). For the $S$/$B$ interactions, $\Delta$ = 6.4 ± 0.5 nm and $\lambda$ is the distance between the two binding pockets, i.e. $\lambda$ ~ 2.5 nm^54^. Hence, for the $S$/$B$ pair, 2-d and 2.5-d are relatively equivalent probably because the bound protein can almost freely rotate when bound to a single $B$.

The case of the interactions between OprM and MexA is very different. The typical dimension of MexA is $\lambda$ ~ 3.5 nm. Our measurements show that for this pair, ${\Delta =k}_{on,2.5d}/{k}_{on,2d}$ = 1.1 ± 0.18 nm. Hence, the 2-d association can be considered about 3 times faster than the 2.5-d association. This difference is key for the association of MexA with OprM because the 2.5-d on-rate, ${k}_{on,2.5d}$ = 131 ± 17 M^–1^.s^–1^ is very small compared to the diffusion-limited collision rate, ~ 10^9^ M^–1^.s^–1^, obtained from the classical Smoluchowski expression^55^, ${k}_{on}=4\pi DR$ where $D$ is the relative translational diffusion constant and $R$ is the sum of the radii of both diffusing objects.

Having both proteins anchored in opposing membranes significantly accelerates bond formation. In addition, the intermembrane distance is critical because we previously showed that OprM and MexA are unable to bind if the membranes are less than 18 nm or more than 22 nm apart^26^, meaning that, for these ranges of intermembrane separations, ${k}_{on,2d}$ = 0. To summarize, studying 2-d *trans* interactions can be useful to control the assembly of protein complexes. Indeed, protein orientation plays a key role for interactions of membrane proteins, as already observed^4^. Adjusting intermembrane distances allows facilitating the formation of specific complexes while hindering others.

### 2.5-d to 3-d association

The dissociation constant between mMexA and OprM in 2.5-d being ${K}_{2.5d}$ = (4.6 ± 1.5)0.10^–7^ M, if both proteins had the same affinity in the 3-d configuration, we should expect them to significantly bind to each other. Indeed, like for the 2-d geometry, at low ${r}_{mAM}$, OprM is in large excess, and we can consider that only a single protein could bind to OprM. If we now suppose that the equilibrium constant ${K}_{3d}$ is equal to ${K}_{2.5d}$ and of the order of 4.6 10^–7^ M, we can calculate the ratio [mMexA]/[${A}_{1}M$] = ${K}_{3d}$/[OprM]_0_ = 0.46/0.8 = 0.58. It means that approximately 35% of labelled mMexA would be free and 65% would be bound to OprM. We should thus see a double exponential recovery curve when performing FRAPP experiments. Since we only observed a single exponential recovery curve, ${K}_{3d}$ must be larger than ${K}_{2.5d}$, suggesting that it is much more difficult for MexA to bind an OprM that is not membrane-anchored.

To confirm our results, we performed the same experiment with native MexA. It diffuses slower in solution in the presence of OprM than alone (Fig. S7 in Supplementary Information) probably because both proteins gather within detergent micelles. This difference may also come from the fact that native MexA has less degrees of freedom in solution because it is self-rotating along with detergent covering its hydrophobic part, and is thus slowed down in its rotation, thus able to interact with OprM.

Focusing now on the interaction between $S$ and biotin in the 3-d geometry, ${\underline{K}}_{3d}$ was measured three decades ago and found to be in the pM range, 2.5.10^–13^ M^56^. More recently ${\underline{K}}_{2.5d}$ was accurately measured using nanowires coated with biotin and found to be slightly lower, 5.6.10^–14^ M^57^. Even though the geometry and freedom of biotin may not be exactly the same as on a membrane, this difference between ${\underline{K}}_{3d}$ and ${\underline{K}}_{2.5d}$ suggests that, as for OprM and MexA, bond formation is facilitated by placing one of the partners on a surface with the correct orientation.

In this article we provided a new method to systematically compare the kinetic parameters of protein association in volume and on membranes. We presented a single parameter involving the on-rates and the distance, $\lambda$, between the binding and anchoring points of the proteins that quantitatively captures which geometry, 2.5-d or 2-d, is more favorable than the other. This parameter, ${k}_{on,2.5d}/\left({k}_{on,2d}\lambda \right)$, globally encapsulates the various degrees of freedom involved in protein interactions including protein orientation that depends on the anchorage to the membrane, fixing them like a keyhole to a door. The orientation of the keyhole makes it easier or more difficult for the key to interact compared to the situation where the keyhole would be moving and self-rotating. We also confirmed the intuitive fact that the off-rates ${k}_{off}$ do not depend on the geometry of protein interactions.

We demonstrated that time resolved FRAPP experiments in a L_3_ phase give precise values for dynamic rates of these associations between soluble and membrane proteins. The time resolution of our technique allows to measure dissociation rates comparable with range obtained by standard techniques^58^. Our approach can be extended to other techniques such as Single Particle Tracking or Fluorescence Correlation Spectroscopy measurements instead of FRAPP.

## Materials and methods

### Proteins

Fluorescein IsoThioCyanate (FITC)-labeled streptavidin was purchased from Interchim and used as received. The twelve-leucine α-helix transmembrane peptide ${L}_{12}$ whose sequence is AKK-${L}_{12}$-GKK was synthesized and biotinylated (${BL}_{12}$) in the Department of Biochemistry and Molecular Genetics (University of Colorado, Denver).

MexA was expressed and purified at 8 mg/mL according to Trépout et al.^59^ in its mature form and its mutant soluble form (with the N-terminal cysteine deleted)^60^. Throughout the manuscript, MexA refers to the palmitoylated version of the protein, that is anchored to the membrane, and mMexA refers to the non-palmitoylated version of the protein.

OprM was expressed and purified at 2 mg/mL as previously described^24,61^.

Depending on the experiments, OprM and MexA could be labeled with FITC (Molecular Probes) following the FluoReporter™ FITC Labeling kit protocol. All protein samples were solubilized in a 50 mM phosphate buffer, pH = 8, containing 100 mM NaCl, 5% (v/v) glycerol and n-octyl-β-D-glucopyranoside (β-OG, Sigma Aldrich) at 0.9% (w/vol).

### ${{\text{L}}}_{3}$ phase

To prepare the ${L}_{3}$ sponge phase^29,62,63^, we mixed a non-ionic surfactant, pentaethylene glycol monododecyl ether (${C}_{12}{E}_{5}$, Sigma Aldrich), β-OG, 5% (v/v) glycerol, and NaCl 100 mM and 50 mM phosphate buffer at pH = 8. The molar ratio β-OG:${C}_{12}{E}_{5}$ was kept constant at 1:7.6. In this phase, bilayers are randomly connected but the membrane volume fraction $\phi =\left({V}_{\upbeta -{\text{OG}}}+{V}_{{C}_{12}{E}_{5}}\right)/{V}_{solution}$ corresponds to a local intermembrane distance ${d}_{w}$. We tuned this fraction to have ${d}_{w}$ = 20 nm^17^ for experiments involving OprM and MexA (since it is the distance that separates in vivo both *Pseudomonas aeruginosa* membranes), or ${d}_{w}$ = 12 nm for experiments with streptavidin and biotinylated peptides, which corresponded to membrane volume fractions $\phi$ of 0.05 and 0.1 respectively. All samples were of 10 μL. The phase is isotropic and stable at room temperature for several weeks. Proteins were added at relatively low concentrations (0.5 to 4 μM) to the surfactant-buffer solution and the sample was vortexed for a few seconds before letting it rest at room temperature for a few minutes prior to proceeding with diffusion measurements. The samples were kept for several weeks at 4 °C and remained stable under these conditions.

### Fluorescence recovery after fringe pattern photobleaching (FRAPP)

The sample was injected into a capillary tube of a 200 μm thickness (VitroCom, Mountain Lakes, New Jersey) which was sealed with wax in order to prevent evaporation. The diffusion coefficient was obtained by performing FRAPP on the sample^11,64^: the laser was focused on a dot of approximately 250 μm of diameter, to insure that the entire thickness of the sample is bleached in order to measure the lateral fluorescence recovery only. The fringe pattern was tuned with various interfringe distances ranging from 10 to 50 μm. The fact that the diffusion is Brownian was verified by using the equation:

3 $$D=\frac{{i}^{2}}{4{\pi }^{2}\tau } ,$$

where $i$ is the interfringe distance and $\tau$ is the recovery time. The fringe pattern has the asset of having a simple geometry: the fluorescence recovery curves were thus easy to fit, $\tau$ being obtained by fitting them with a simple exponential if there was one diffusing regime, or a double exponential if the object (lipid, peptide or protein) was diffusing at two different speeds, or if there were two objects diffusing differently. FRAPP measurements on the ${L}_{3}$ phase are accurate: the phase being isotropic and formed on a large volume, bleaching experiments are automatized in order to sum up at least 10 times the fluorescence recovery curves and reduce the noise. The ${L}_{3}$ phase is volumetric, so there is no problem of fluorescence loss or acquisition bleaching. In total, we were able to measure the diffusion coefficient of an object in the sponge phase with an accuracy of 2 to 5%.

### Supplementary Information

Supplementary Information.

## Data Availability

The datasets generated and analyzed during the current study are available from the corresponding author on reasonable request.
