# Author Correction: Kinetic study of membrane protein interactions: from three to two dimensions

**DOI:** 10.1038/s41598-024-58201-9

**Published:** 2024-04-08

**Authors:** Vladimir Adrien, Myriam Reffay, Nicolas Taulier, Alice Verchère, Laura Monlezun, Martin Picard, Arnaud Ducruix, Isabelle Broutin, Frédéric Pincet, Wladimir Urbach

**Affiliations:** 1 Laboratoire de Physique de l’École normale superieure, École Normale Supérieure, Université Paris Sciences et Lettres, CNRS, Sorbonne Université, Université Paris Cité, F-75005 Paris, France; 2 Department of Infectious Diseases, Avicenne Hospital, AP-HP, Université Sorbonne Paris Nord, Bobigny, France; 3 Université Paris Cité, Inserm UMR-S 1266, Institute of Psychiatry and Neuroscience of Paris (IPNP), Paris, France; 4 Laboratoire Matière et Systèmes Complexes, UMR 7057, CNRS, Université de Paris Cité, 75205 Paris Cedex 13, France; 5 Sorbonne Université, CNRS, INSERM, Laboratoire d′Imagerie Biomédicale-LIB, 75006 Paris, France; 6 Laboratoire CiTCoM, Faculté de Santé, Université Paris Cité, CNRS, 75006 Paris, France; 7 Université Paris Cité, CNRS, Expression Génétique Microbienne, Institut de Biologie Physico-Chimique, Paris, France; 8 Université Paris Cité, Laboratoire de Biologie Physico-Chimique des Protéines Membranaires CNRS UMR7099, 75005 Paris, France; 9 Institut de Biologie Physico-Chimique, Fondation Edmond de Rothschild, 75005 Paris, France

Correction to: *Scientific Reports* 10.1038/s41598-023-50827-5, published online 09 January 2024

Myriam Reffay was omitted from the author list in the original version of this Article.

Additionally, Vladimir Adrien and Myriam Reffay were omitted as equally contributing authors.

Finally, the Author Contributions section was incorrect.

“V.A., N.T., F.P. and W.U. made substantial contributions to the conception and design of the work. V.A., N.T., A.V., L.M., M.P., F.P. and W.U. did the experiments. V.A., N.T., F.P. and W.U. participated in the interpretation and visualization of data. V.A. and N.T. have drafted the work. F.P. and W.U. supervised the work. All authors reviewed the manuscript and have approved the submitted version.”

now reads:

“V.A., M.R., N.T., F.P. and W.U. made substantial contributions to the conception and design of the work. V.A., M.R., N.T., A.V., L.M., M.P., F.P. and W.U. did the experiments. V.A., M.R., N.T., F.P. and W.U. participated in the interpretation and visualization of data. F.P. and W.U. supervised the work. V.A., F.P. and W.U. wrote the manuscript. All authors reviewed the manuscript and have approved the submitted version.”

The original Article and accompanying Supplementary Information file have been corrected.

