# Supplementary material for: Kinetic study of membrane protein interactions: from three to two dimensions
Source: Sci Rep. 2024 Jan 9;14:882. doi: 10.1038/s41598-023-50827-5 (PMC10776792; doi:10.1038/s41598-023-50827-5)
Supplement: Supplementary file 1 — Supplementary Information. [file 41598_2023_50827_MOESM1_ESM.docx]

Supplementary Information for

Kinetic study of membrane protein interactions: from three to two dimensions

Vladimir Adrien^1,2,3,﻿^*^,†^, Myriam Reffay^4,†^, Nicolas Taulier^5^, Alice Verchère^6^, Laura Monlezun^7^, Martin Picard^8,9^, Arnaud Ducruix^6^, Isabelle Broutin^6^, Frédéric Pincet^1,^*, Wladimir Urbach^1,5,^*

**Affiliations**

^1^ Laboratoire de Physique de l’École normale supérieure, ENS, Université PSL, CNRS, Sorbonne Université, Université Paris Cité, F-75005 Paris, France.

^2^ AP-HP, Department of Infectious Diseases, Avicenne Hospital, Paris Nord Sorbonne Université, Bobigny, France.

^3^ Université Paris Cité, INSERM, UMR-S 1266, Institut de Psychiatrie et Neurosciences de Paris, Paris, France.

^4^ Laboratoire Matière et Systèmes Complexes, UMR 7057, CNRS and Université Paris Cité, 75205 Paris Cedex 13, France.

^5^ Sorbonne Université, CNRS, INSERM, Laboratoire d'Imagerie Biomédicale, LIB, F-75006 Paris, France.

^6^ Université Paris Cité, CNRS, CiTCoM, F-75006 Paris, France

^7^ Université Paris Cité, CNRS, Expression Génétique Microbienne, Institut de Biologie Physico-Chimique, Paris, France

^8^ Université Paris Cité, Laboratoire de Biologie Physico-Chimique des Protéines Membranaires CNRS UMR7099, Paris 75005, France

^9^ Institut de Biologie Physico-Chimique, Fondation Edmond de Rothschild, Paris 75005, France.

^†^ Equal contribution

* Corresponding authors: [vladimir.adrien@aphp.fr](mailto:vladimir.adrien@aphp.fr), [frederic.pincet@ens.fr](mailto:frederic.pincet@ens.fr), [urbach@lps.ens.fr](mailto:urbach@lps.ens.fr)

1. Quenching and 2-d affinity of MexA and OprM

Because of the quenching between dyes in the $A_{2}M$ complex, the conversion between the fluorescence intensities and the concentrations can be written as:

$$\left\{ \begin{aligned} I_{F}=\gamma_{F}a[MexA] \\ I_{A_{1}M}= \gamma_{F}a[A_{1}M] \\ I_{A_{2}M}= {2\gamma}_{F}\eta a[A_{2}M] \end{aligned} (S1) \right.$$

Where $\gamma_{F}$ is the proportionality coefficient that relates the concentration of MexA to the observed fluorescence, $\eta$ is an unknown corrective factor due to the quenching ($\eta$ < 1), and $a$ is the surface of the illuminated region of the bilayer. [ ] notation is for surface concentration.

We hypothesize that $\gamma_{F}$ is the same for all species and will verify it later.

The two corresponding reactions are:

$$\left\{ \begin{aligned} MexA+OprM \underset{\leftrightarrow}{K_{2d}}A_{1}M \\ MexA+A_{1}M \underset{\leftrightarrow}{K_{2d}}A_{2}M \end{aligned} (S2) \right.$$

We assume that the two reactions have the same 2-d affinity (non-cooperative reactions):

$$K_{2d}=\frac{2\left[ MexA \right][OprM]}{[A_{1}M]}=\frac{\left[ MexA \right][A_{1}M]}{2[A_{2}M]} (S3)$$

We can easily solve this system with the conditions at the beginning of the experiment ([MexA]_0_ = $r_{AM}$[OprM]_0_ and [$A_{1}M$] = [$A_{2}M$] = 0), where [OprM]_0_ and [MexA]_0_ are the initial concentrations of OprM and MexA, and with the conservation of MexA:

$$r_{AM}{[OprM]}_{0}={[MexA]}_{0}=[MexA]+[A_{1}M]+2[A_{2}M] (S4)$$

Before the stoichiometric ratio is reached ($r_{AM}$ < 2), almost each MexA protein interacts with OprM as shown by the fact that $I_{F}$ ≈ 0 (Figure 1C), thus [MexA] ≈ 0.

We can write the concentration of each species at t=0 and at the equilibrium (Table S1):

|  | [MexA] | [OprM] | [$A_{1}M$] | [$A_{2}M$] |
| --- | --- | --- | --- | --- |
| t=0 | $r_{AM}[{OprM]}_{0}$ | $[{OprM]}_{0}$ | 0 | 0 |
| Equilibrium | $r_{AM}[{OprM]}_{0}-x-y$ | $[{OprM]}_{0}-x$ | $x-y$ | $y$ |

Table S1. Concentration of OprM, MexA, $\boldsymbol{A}_{\boldsymbol{1}}\boldsymbol{M}$ and $\boldsymbol{A}_{\boldsymbol{2}}\boldsymbol{M}$ at t=0 and at the equilibrium. $\boldsymbol{r}_{\boldsymbol{AM}}$ is the molar ratio between MexA and OprM. $\mathbf{y}$is the concentration of $\boldsymbol{A}_{\boldsymbol{2}}\boldsymbol{M}$ at the equilibrium. $\mathbf{x-y}$ is the concentration of $\boldsymbol{A}_{\boldsymbol{1}}\boldsymbol{M}$ at the equilibrium.

From Equation (S3), we now can write:

$$K_{2d}= \frac{2(r_{AM}[{OprM]}_{0}-x-y)([{OprM]}_{0}-x)}{x-y}=\frac{(r_{AM}[{OprM]}_{0}-x-y)(x-y)}{2y} (S5)$$

where $y$is the concentration of $A_{2}M$ at the equilibrium and $x-y$ is the concentration of $A_{1}M$ at equilibrium.

Then:

$$\frac{{[OprM]}_{0}-x}{x-y}=\frac{x-y}{4y} (S6)$$

When $r_{AM}$ << 2, [MexA] ≈ 0, thus:

$$x= r_{AM}[{OprM]}_{0}-y (S7)$$

From Equation (S6) and (S7):

$$\left[ A_{2}M \right]= y=\frac{{r_{AM}}^{2}[{OprM]}_{0}}{4} (S8)$$

Finally, from Equation (S7) and (S8),

$$\left[ A_{1}M \right]=x-y=[{OprM]}_{0}r_{AM}\left( 1-\frac{r_{AM}}{2} \right) \approx r_{AM}[{OprM]}_{0} (S9)$$

From Equation (S3):

$$\left[ MexA \right]={2K}_{2d}\frac{\left[ A_{2}M \right]}{\left[ A_{1}M \right]}=K_{2d}\frac{r_{AM}}{2} \left( S10 \right)$$

When $r_{AM}$ >> 2, we can this time consider [OprM] ≈ 0, thus $x= [{OprM]}_{0}$.

From Equation (S6), we deduce $y= [{OprM]}_{0}$.

Thus we can write:

$$\left[ MexA \right]=r_{AM}[{OprM]}_{0}-x-y=(r_{AM}-2)[{OprM]}_{0}\approx r_{AM}[{OprM]}_{0} (S11)$$

As expected, $I_{F}$ exhibits a linear behaviour for both regimes ($r_{AM}$ < 2 and $r_{AM}$ > 2). The ratio between the two slopes can be written:

$$\frac{\left[ MexA \right]_{r>2}}{\left[ MexA \right]_{r<2}}\approx\frac{{2 r}_{AM}[{OprM]}_{0}}{K_{2d}r_{AM}}=\frac{2[{OprM]}_{0}}{K_{2d}} (S12)$$

We deduce $K_{2d}={2[{OprM]}_{0}s_{1}}/{s_{2}}$ where $s_{1}$ is the slope of $I_{F}$ when $r_{AM}$ < 2 and $s_{2}$ is the slope of $I_{F}$ when $r_{AM}$ > 2. Linear fits give $s_{1}$ = 3.90 ± 0.27 and $s_{2}$ = 192.25 ± 5.45. We thus obtain$K_{2d}$ = 2*2.10^-13^*3.7147/196.68 = (8.1± 0.61) 10^-15^ mol.dm^-2^.

S2. Temporal evolution of the ratio ${\boldsymbol{I}_{\boldsymbol{B}}}/{\boldsymbol{(}\boldsymbol{I}_{\boldsymbol{F}}\boldsymbol{+}\boldsymbol{I}_{\boldsymbol{B}}}\boldsymbol{)}$

The temporal evolution of the ratio ${I_{B}}/{(I_{F}+I_{B}})$ (Figure 1E) shows that the proportion of bound MexA proteins decreases with time. We can write $I_{B}=I_{Bo}e^{-k_{off}t}$ and $I_{F}=I_{Fo}+(I_{F\infty}-I_{Fo})(1-e^{-k_{off}t})$ where $I_{Bo}$ and $I_{Fo}$ are intensity values of $I_{B}$ and $I_{F}$ respectively and $I_{F\infty}$ is the intensity value of $I_{F}$ after a long time.

We also consider $I_{F}+I_{B}=I_{Fo}+I_{Bo}$ as the number of fluorophores is constant. Thus:

$$\frac{I_{B}}{I_{F}+I_{B}}=\frac{I_{Bo}}{I_{Fo}+I_{Bo}}e^{-k_{off}t} (S13)$$

A fit of the experimental data gives $k_{off}$ = (1.0 ± 0.13) 10^-4^ s^-1^. It is an order of magnitude for $k_{off}$.

The reactions to consider are:

$$\left\{ \begin{aligned} {MexA}^{*}+OprM\leftrightarrow A_{1}^{*}M \\ {MexA}^{*}+A_{1}^{*}M \leftrightarrow A_{2}^{*}M \\ MexA+ A_{1}^{*}M \leftrightarrow A^{*}AM \\ MexA+OprM \leftrightarrow A_{1}M \\ MexA+ A_{1}M\leftrightarrow A_{2}M \\ {MexA}^{*}+A_{1}M \leftrightarrow A^{*}AM \end{aligned} (S14) \right.$$

The kinetic equations are: where the sign * refers to labelled proteins. All these reactions have the same dissociation and association rate constants $k_{off}$ and $k_{on,2d}$ = ${k_{off}}/{K_{2d}}$.

$$\left\{ \begin{aligned} {d\left[ MexA \right]}/{dt}=-k_{on,2d}\left[ MexA \right]\left( \left[ OprM \right]+\left[ A_{1}M \right]+\left[ A_{1}^{*}M \right] \right)+k_{off}\left( \left[ A_{1}M \right]+2\left[ A_{2}M \right]+\left[ AA^{*}M \right] \right) \\ {d\left[ MexA^{*} \right]}/{dt} =-k_{on,2d}\left[ {MexA}^{*} \right]\left( \left[ OprM \right]+\left[ A_{1}M \right]+\left[ A_{1}^{*}M \right] \right)+k_{off}\left( \left[ A_{1}^{*}M \right]+2\left[ A_{2}^{*}M \right]+\left[ AA^{*}M \right] \right) \\ {d\left[ A_{1}M \right]}/{dt}= k_{on,2d}\left( \left[ MexA \right]\left[ OprM \right]-\left[ MexA \right]\left[ A_{1}M \right]-\left[ {MexA}^{*} \right]\left[ A_{1}M \right] \right)-k_{off}\left( \left[ A_{1}M \right]-2\left[ A_{2}M \right]-\left[ AA^{*}M \right] \right) \\ {d\left[ A_{1}^{*}M \right]}/{dt}=k_{on,2d}(\left[ {MexA}^{*} \right]\left[ OprM \right]-\left[ MexA^{*} \right]\left[ A_{1}M \right]-\left[ MexA \right]\left[ A_{1}^{*}M \right]-k_{off}\left( \left[ A_{1}^{*}M \right]-2\left[ A_{2}^{*}M \right]-\left[ AA^{*}M \right] \right) \\ {d\left[ A_{2}M \right]}/{dt}=k_{on,2d}\left[ MexA \right]\left[ A_{1}M \right]- {2k}_{off}\left[ A_{2}M \right] \\ {d\left[ A_{2}^{*}M \right]}/{dt} = k_{on,2d}\left[ {MexA}^{*} \right]\left[ A_{1}^{*}M \right]- {2k}_{off}\left[ A_{2}^{*}M \right] \\ {d\left[ AA^{*}M \right]}/{dt}=k_{on,2d}(\left[ {MexA}^{*} \right]\left[ A_{1}M \right]+\left[ MexA \right]\left[ A_{1}^{*}M \right])- {2k}_{off}\left[ AA^{*}M \right] \end{aligned} (S15) \right.$$

Initially, we know the quantity of labelled MexA (free or bound) since we know $K_{2d}$. We thus have [MexA^*^] = 3.3 10^-14^ mol.dm^-2^, [$A_{1}^{*}M$] = 2.6 10^-14^ mol.dm^-2^, [$A_{2}^{*}M$] = 1.7 10^-13^ mol.dm^-2^, and we deduce [OprM] = 3.8 10^-15^ mol.dm^-2^. Furthermore, the concentration of free unlabelled proteins is [MexA] = 40 10^-12^ mol.dm^-2^. The initial concentrations of other species are null.

We solved the system numerically to obtain the temporal evolution of labelled components concentrations (Figure S1).

~~
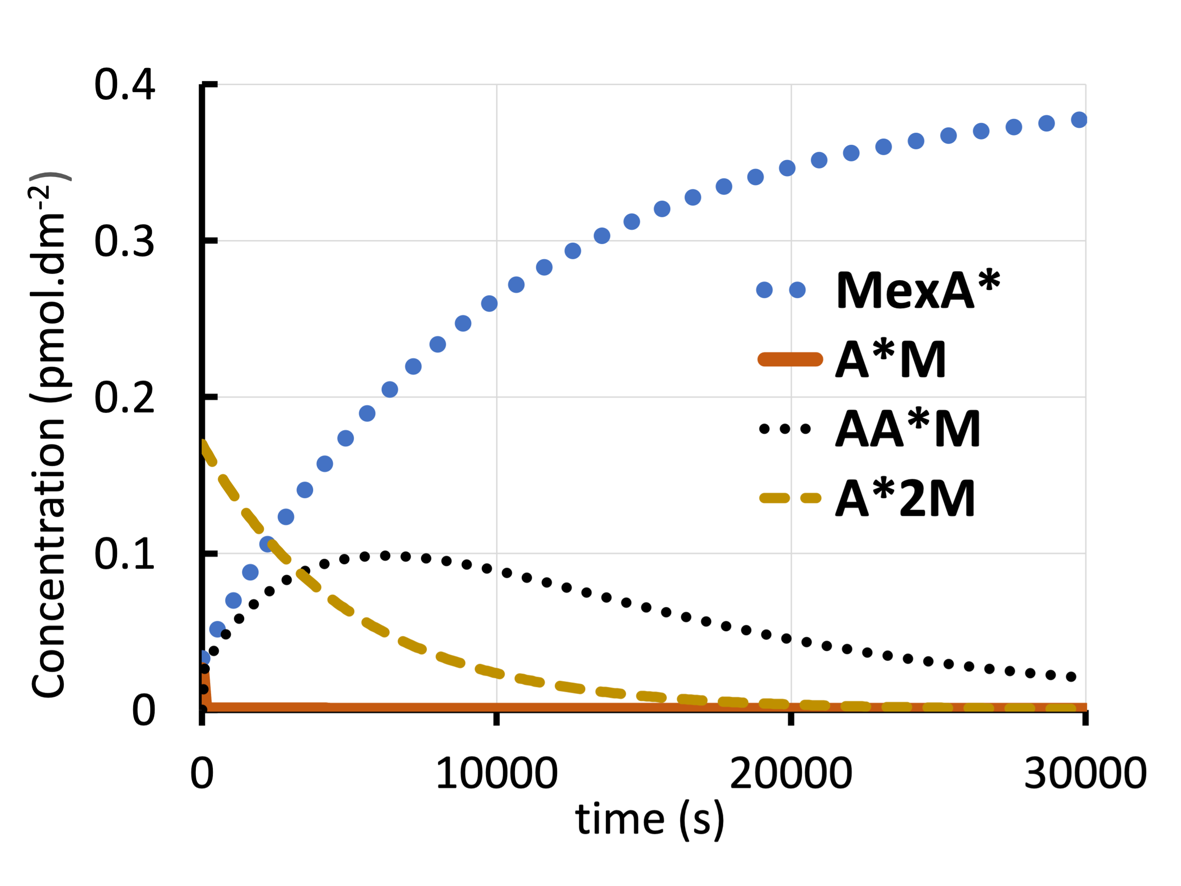
~~

Fig. S1. Numerical solution of the system Equation (S19).

We see that initially [MexA^*^] and [$AA^{*}M$] increase whereas [$A_{1}^{*}M$] and [$A_{2}^{*}M$] decrease. Indeed, each labelled component will be replaced by an unlabelled component.

S3. Determination of the surface concentration of proteins in the L_3_ phase

The area of the monolayer is given by:

$$a=\frac{V\phi\sigma\rho\mathcal{N}_{a}}{M_{C_{12}E_{5}}} (S16)$$

Where $M_{C_{12}E_{5}}$ is the molar mass of surfactant ($M_{C_{12}E_{5}}$ = 406.6 g.mol^-1^), $V$ is the volume of the solution, $\phi$ the chosen volume fraction of membrane (here $\phi$ = 0.05 for experiments with MexA and OprM and $\phi$ = 0.1 for experiments with streptavidin and biotin), $\sigma$ the are per polar head of surfactant ($\sigma$ = 0.42 nm^2^)^1^, $\rho$ is the volume mass of surfactant ($\rho$ = 0.967 g/mL) and $\mathcal{N}_{a}$ is the Avogadro constant.

Since sample volumes were of 10 µL, we obtain $a$ = 30 dm^2^ for OprM-MexA experiments and $a$ = 60 dm^2^ for streptavidin-biotin experiments.

The surface concentration $c_{surf}$ of protein is deduced from the quantity of protein $n_{prot}$ added in the sample:

$$c_{surf}=\frac{n_{prot}}{a}=\frac{c_{prot}M_{C_{12}E_{5}}}{\phi\sigma\rho\mathcal{N}_{a}} (S17)$$

We added a volume concentration {MexA} = 3.2 µM which gives a surface concentration [MexA] ~ 1 pmol.dm^-2^.

S4. Proportionality of $\boldsymbol{B}$, $\boldsymbol{B}_{\boldsymbol{1}}\boldsymbol{S}$ and $\boldsymbol{B}_{\boldsymbol{2}}\boldsymbol{S}$ concentrations with their fluorescence intensities

We introduced a concentration of streptavidin of 9.7 10^-7^ M and varied the concentration of $B$. If we write $r_{BS}={n_{B_{0}}}/{n_{S_{0}}}$ where $n_{B_{0}}$ and $n_{S_{0}}$ are respectively the initial number of $B$ and streptavidin in the sample, then the conservation of $B$ is written:

$$n_{B_{0}}=r_{BS}n_{S_{0}}=n_{B}+n_{B_{1}S}+{2n}_{B_{2}S} (S18)$$

Where $n_{B_{1}S}$ and $n_{B_{2}S}$ are this time the number of $B_{1}S$ and $B_{2}S$ formed. If $r_{BS}$ << 1 we can consider $n_{B}$ ~ 0. If we assume that the fluorescence intensities of $B_{1}S$ and $B_{2}S$ have the same proportionality coefficient $\gamma$ to their concentration, then:

$$n_{B_{0}} =\frac{1}{\gamma}\left( I_{1S}+2I_{2S} \right)=r_{BS}n_{S_{0}} (S19)$$

The variation of $I_{1S}+2I_{2S}$ with $r$ is in Figure S2.


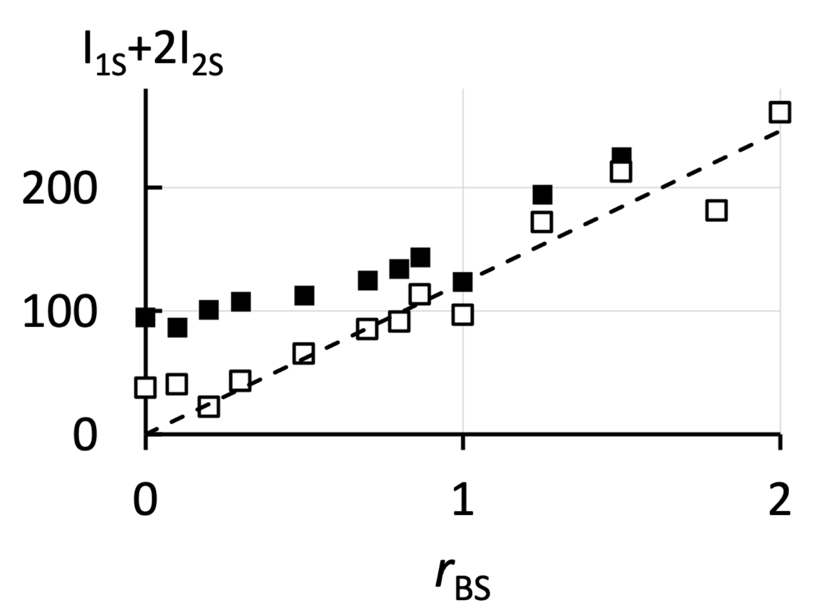


Fig. S2. The variation of $\boldsymbol{I}_{\boldsymbol{1}\boldsymbol{S}}\boldsymbol{+2}\boldsymbol{I}_{\boldsymbol{2}\boldsymbol{S}}$ with $\mathbf{r}_{\mathbf{BS}}$ is represented with black squares. If we consider $\boldsymbol{I}_{\boldsymbol{1}\boldsymbol{S}}\boldsymbol{=}\boldsymbol{I}_{\boldsymbol{1}\boldsymbol{S, measured}}\boldsymbol{-\beta}\boldsymbol{I}_{\boldsymbol{0}\boldsymbol{S}}$, the corrected values are represented with white squares and show a linear variation (Equation (S19)).

We see that the relation is not linear. Indeed, on samples with streptavidin alone, the fluorescence recovery exhibits a double exponential behaviour, with the presence of a slow diffusion of the order of 2.8 µm^2^.s^-1^, showing that a part of the protein diffuses on the membrane. This membrane attached fraction is supposed to be proportional to the free streptavidin bound to $B$ at low $r_{BS}$ with a factor $\beta$ between both that we measured at 3 ± 0.5.

If we now consider $I_{1S}= I_{1S, measured}-\beta I_{0S}$, we obtain the corrected values represented in Figure S2. It is now a linear variation with a slope $\gamma n_{S_{0}}$ = 141 ± 3.

Next, we can write the conservation of streptavidin:

$$n_{S_{0}}=n_{0S}+n_{1S}+n_{2S} (S20)$$

Where $n_{0S}$, $n_{1S}$ and $n_{2S}$ are respectively the quantity of free streptavidin, and streptavidin bound to one or two $B$. We can now write:

$$n_{S_{0}}=\frac{1}{\gamma_{v}}I_{0S}+\frac{1}{\gamma}\left( I_{1S}+I_{2S} \right) (S21)$$

Where $\gamma_{v}$ is the proportionality coefficient between $I_{0S}$ and $n_{0S}$. Since the streptavidin concentration is constant, we check the variation of $I_{1S}+I_{2S}$ with $I_{1S}$ with the corrected value of $I_{1S}$. We suppose that the correction factor on $I_{0S}$ is negligible. The obtained results confirm a proportionality, as shown in Figure S3.


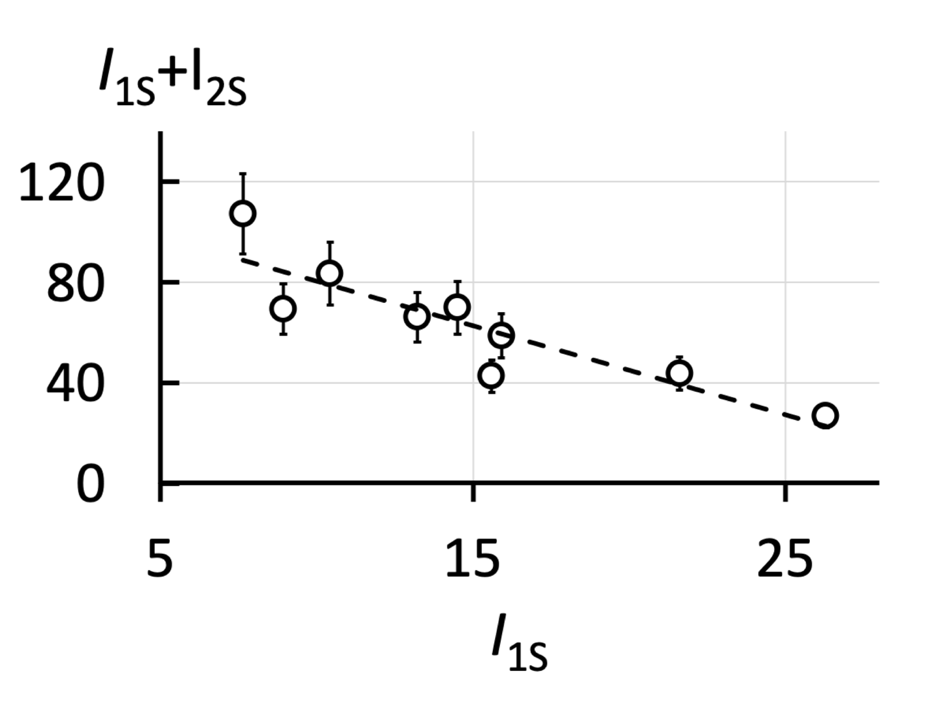


Fig. S3. Variation of $\boldsymbol{I}_{\boldsymbol{1}\boldsymbol{S}}\boldsymbol{+}\boldsymbol{I}_{\boldsymbol{2}\boldsymbol{S}}$ with the corrected value of $\boldsymbol{I}_{\boldsymbol{1}\boldsymbol{S}}$. The obtained results confirm a proportionality (Equation (S21)).

S5. Concentrations of $\boldsymbol{B}$, $\boldsymbol{B}_{\boldsymbol{1}}\boldsymbol{S}$ and $\boldsymbol{B}_{\boldsymbol{2}}\boldsymbol{S}$

The reactions to consider are:

$$\left\{ \begin{aligned} B+S\underset{\to}{\underline{k}_{on,2.5d}}B_{1}S \\ B+B_{1}S \underset{\to}{\underline{k}_{on,2d}} B_{2}S \end{aligned} \right. (S22)$$

We assume $\underline{k}_{off}\approx0$ thus the equilibrium is reached when $\left[ B \right]=0$. Both reactions of Equation (S27) are simultaneous. Kinetic equations of the system give:

$$\left\{ \begin{aligned} {d\left[ B \right]}/{dt}= -\underline{k}_{on,2.5d}\left[ B \right]\left\{ S \right\}_{eff}-\underline{k}_{on,2d}\left[ B \right]\left[ B_{1}S \right] \\ {d\left[ B_{1}S \right]}/{dt}= \underline{k}_{on,2.5d}\left[ B \right]\left\{ S \right\}_{eff}-\underline{k}_{on,2d}\left[ B \right]\left[ B_{1}S \right] \\ {d\left[ B_{2}S \right]}/{dt}=\underline{k}_{on,2d}\left[ B \right]\left[ B_{1}S \right] \end{aligned} (S23) \right.$$

Where $a$ = 60 dm^2^ is the surface of the monolayer given by Equation (S16), and $\left\{ S \right\}_{eff}=\left\{ S \right\}V/V_{eff}$. The notation {} is for volume concentration. $V$ = 10 µL is the volume of the sample and $V_{eff}$ the effective aqueous volume between bilayers accessible to streptavidin.

$V_{eff}=V-a\left( e/2+r_{H} \right)$ where $e/2$ = 1.6 nm is the monolayer thickness^2^ and $r_{H}$ = 2.5 nm is the hydrodynamic radius of streptavidin. Thus $V_{eff}$= 7.54 µL. $V_{eff}$is represented on Figure S4. The protein cannot be at a distance from the bilayer smaller than its hydrodynamic radiu*s* $r_{H}$.


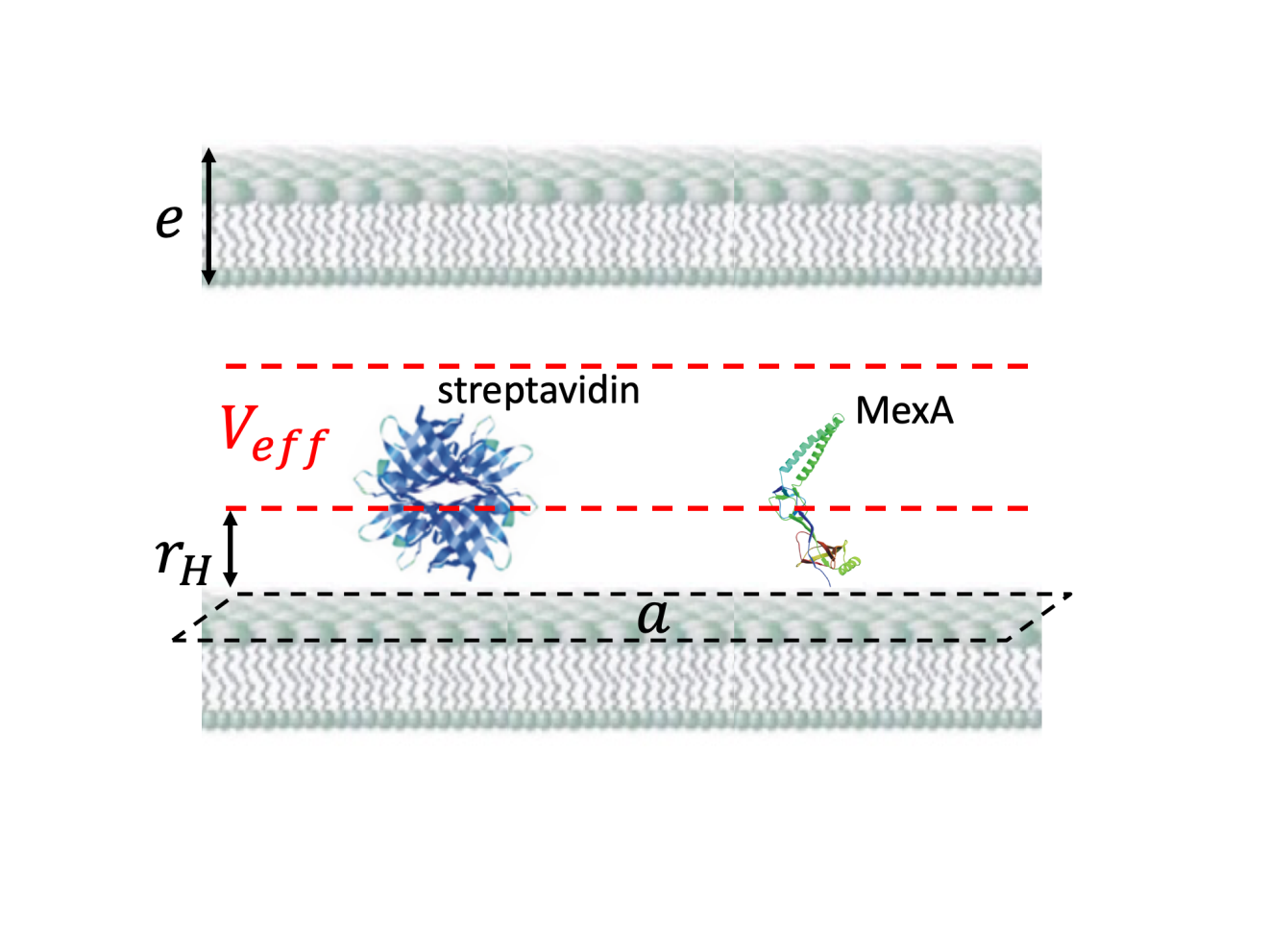


Fig. S4. Sketch representing the effective diffusion volume $\boldsymbol{V}_{\boldsymbol{eff}}$ for the mutant soluble protein mMexA and streptavidin. The protein cannot be at a distance from the bilayer smaller than its hydrodynamic radius $\boldsymbol{r}_{\boldsymbol{H}}$. Thus $\boldsymbol{V}_{\boldsymbol{eff}}\boldsymbol{=V-a(}\boldsymbol{e}/\boldsymbol{2}\boldsymbol{+}\boldsymbol{r}_{\boldsymbol{H}}\boldsymbol{)}$ where $\boldsymbol{V}$ is the volume of the sample, $\boldsymbol{e}$ is the bilayer thickness, and $\boldsymbol{a}$ the surface of the monolayer.

To simplify Equation (S23), we made some assumptions: at small $r_{BS}$, since streptavidin is in excess, we can consider that its bound fraction is negligible compared to $\left\{ S \right\}_{eff}$, thus $\left\{ S \right\}_{eff}=\left\{ S_{0} \right\}_{eff}=\left\{ S_{0} \right\}V/V_{eff}$ = 1.33 µM. Equation (S23) then gives:

$$\left\{ \begin{aligned} {d\left[ B \right]}/{dt}= -\underline{k}_{on,2.5d}\left[ B \right]\left\{ S_{0} \right\}_{eff} \\ {d\left[ B_{1}S \right]}/{dt}= \underline{k}_{on,2.5d}\left[ B \right]\left\{ S_{0} \right\}_{eff} \\ {d\left[ B_{2}S \right]}/{dt}=\underline{k}_{on,2d}\left[ B \right]\left[ B_{1}S \right] \end{aligned} \left( S24 \right) \right.$$

From the first equation, we deduce:

$$\left[ B \right]= \left[ B_{0} \right]exp({-t}/{\tau)} (S25)$$

Where $\tau=1/\underline{k}_{on,2.5d}\left\{ S_{0} \right\}_{eff}$ and $\left[ B_{0} \right]$ is the biotin initial surface concentration. From Equation (S24) and Equation (S25), we write:

$${d\left[ B_{1}S \right]}/{dt}= \frac{\left[ B_{0} \right]}{\tau}exp({-t}/{\tau)} (S26)$$

From which we deduce:

$$\left[ B_{1}S \right]= \left[ B_{0} \right]\left( 1-exp({-t}/{\tau)} \right) (S27)$$

Finally, from Equation (S24) and (S25) and (S27), we write:

$${d\left[ B_{2}S \right]}/{dt}=\underline{k}_{on,2d}\left[ B_{0} \right]^{2}exp({-t}/{\tau)}\left( 1-exp({-t}/{\tau)} \right) (S28)$$

Since $\left[ B_{2}S \right]_{0}$ ~ 0, we deduce:

$$\left[ B_{2}S \right]=\underline{k}_{on,2d}\left[ B_{0} \right]^{2}\tau\left( \frac{1}{2}-exp({-t}/{\tau)}+\frac{1}{2}exp({-2t}/{\tau)} \right) (S29)$$

At steady-state (time scale of several hours >> $\tau$), Equation (S25), (S27) and (S29) give:

$$\left\{ \begin{aligned} \left[ B \right]=0 \\ \left[ B_{1}S \right]=\left[ B_{0} \right] \\ \left[ B_{2}S \right]=\frac{\underline{k}_{on,2d}\left[ B_{0} \right]^{2}}{2\underline{k}_{on,2.5d}\left\{ S_{0} \right\}_{eff}} \end{aligned} (S30) \right.$$

Equation (S30) gives $\left[ B_{2}S \right]/\left[ B_{1}S \right]={\underline{k}_{on,2d}r_{BS}V_{eff}}/{2a\underline{k}_{on,2.5d}}$ at small $r_{BS}$ indicating that ${I_{2S}}/{I_{1S}}$should vary linearly with a slope ${\underline{k}_{on,2d}V_{eff}}/{2a\underline{k}_{on,2.5d}}$. The data are indeed linear (Figure 2B) and lead to ${\underline{k}_{on,2.5d}}/{\underline{k}_{on,2d}}$ = 6.4 ± 0.4 nm.

S6. Predicted variation of ${\boldsymbol{I}_{\boldsymbol{2}\boldsymbol{S}}}/{\boldsymbol{I}_{\boldsymbol{1}\boldsymbol{S}}}$

We can write the surface concentrations $\left[ B_{1}S \right]={n_{B_{1}S}}/a$ and $\left[ B_{2}S \right]={n_{B_{2}S}}/a$ where $a$ = 60 dm^2^ is given by Equation (S16).

The conservation of the total number of streptavidin molecules can be written:

$$n_{S_{0}}=V\left\{ S \right\}+a\left( \left[ B_{1}S \right]+\left[ B_{2}S \right] \right) (S31)$$

which can be written:

$$\left\{ S \right\}_{0}=\left\{ S \right\}+\frac{a}{V}\left( \left[ B_{1}S \right]+\left[ B_{2}S \right] \right) (S32)$$

Hence:

$${d\left\{ S \right\}}/{dt}+\frac{a}{V}\left( {d\left[ B_{1}S \right]}/{dt}+{d\left[ B_{2}S \right]}/{dt} \right)=0 \left( S33 \right)$$

The conservation of the total number of biotin molecules can be written:

$$n_{B_{0}}=r_{BS}\left\{ S \right\}_{0}=a\left( \left[ B \right]+\left[ B_{1}S \right]+2\left[ B_{2}S \right] \right) (S34)$$

Hence:

$${d\left[ B \right]}/{dt}+{d\left[ B_{1}S \right]}/{dt}+{2d\left[ B_{2}S \right]}/{dt}=0 \left( S35 \right)$$

Equation (S23), (S33) and (S35) give:

$$\left\{ \begin{aligned} {d\left[ B \right]}/{dt}= -\frac{V}{V_{eff}}\underline{k}_{on,2.5d}\left[ B \right]\left\{ S \right\}-\underline{k}_{on,2d}\left[ B \right]\left[ B_{1}S \right] \\ {d\left\{ S \right\}}/{dt}=-\frac{a}{V_{eff}}\underline{k}_{on,2.5d}\left[ B \right]\left\{ S \right\} \\ {d\left[ B_{1}S \right]}/{dt}= \frac{V}{V_{eff}}\underline{k}_{on,2.5d}\left[ B \right]\left\{ S \right\}_{eff}-\underline{k}_{on,2d}\left[ B \right]\left[ B_{1}S \right] \\ {d\left[ B_{2}S \right]}/{dt}=\underline{k}_{on,2d}\left[ B \right]\left[ B_{1}S \right] \end{aligned} (S36) \right.$$

We plotted the predicted variations of ${I_{2S}}/{I_{1S}}$ (Figure 2A), $I_{1S}$ and $I_{2S}$ (Figure S5) over a wider range of $r_{BS}$ using ${\underline{k}_{on,2.5d}}/{\underline{k}_{on,2d}}$ measured for small $r_{BS}$. The resulting curves nicely fit the experimental data, confirming the validity of our approach.


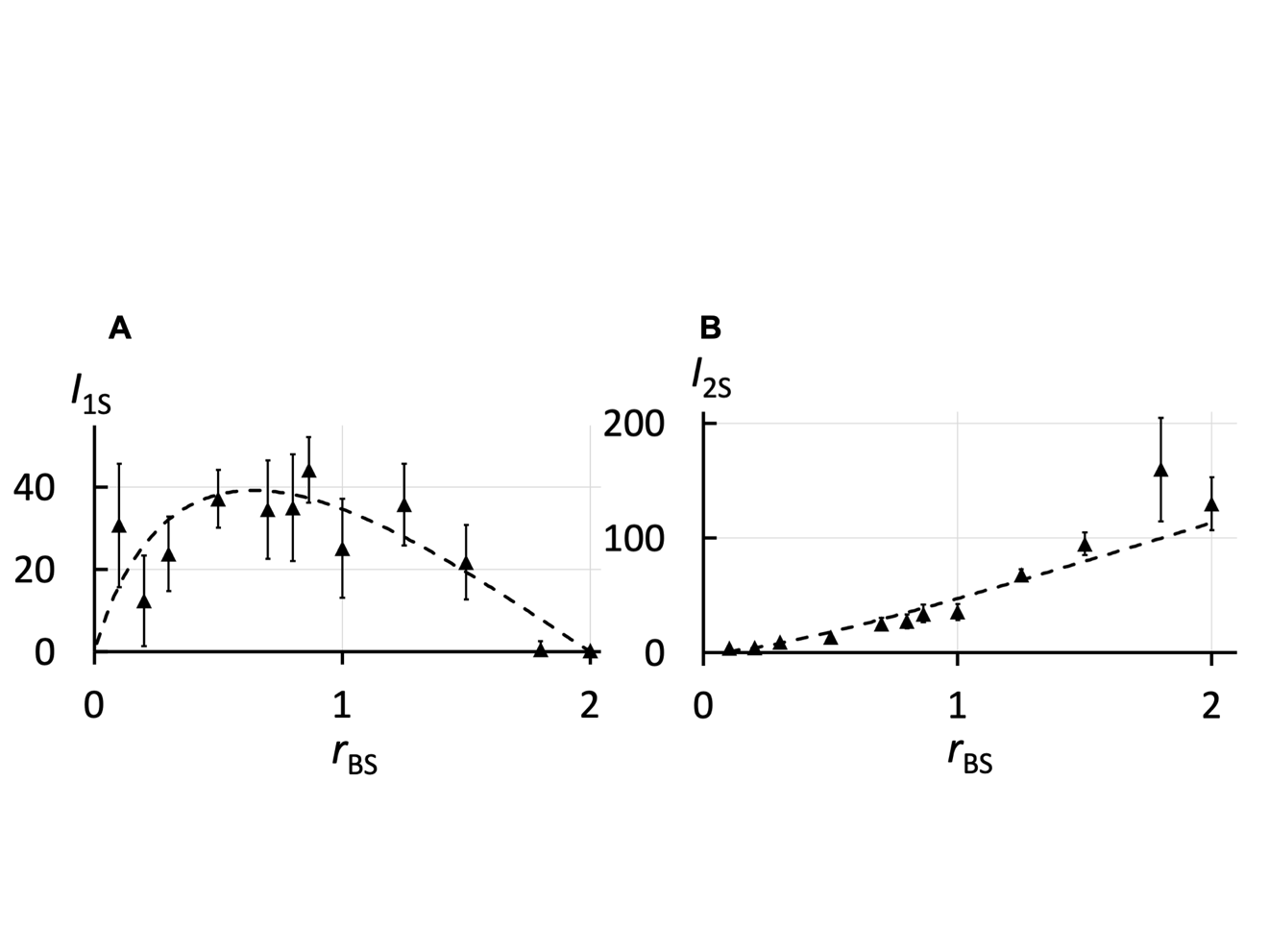


Fig. S5. (A) Variations of the intensities $\boldsymbol{I}_{\boldsymbol{1}\boldsymbol{S}}$ corresponding to the $\boldsymbol{S}$ linked to one $\boldsymbol{B}$ are also compared to the calculations (- -) (Equation (S36)). (B) Variations of the intensities $\boldsymbol{I}_{\boldsymbol{2}\boldsymbol{S}}$ corresponding to the $\boldsymbol{S}$ linked to two $\boldsymbol{B}$ are also compared to the calculations (- -) (Equation (S36)).

**S7. 2.5-d OprM-MexA association kinetics**

The intensities $I_{mF}$ and $I_{mB}$ are proportional to the number of the free and bound mMexA and can be written

$$\left\{ \begin{aligned} I_{mF}= \gamma_{mF}V_{eff}\left\{ mMexA \right\} \\ I_{mB}= \gamma_{mB}a\left[ mA_{1}M \right] \end{aligned} \right. (S37)$$

${mA}_{1}M$ is diffusing on the surface *a* of the bilayers, mMexA is a soluble protein diffusing in the effective aqueous volume $V_{eff}$ between bilayers accessible to the center of mass of mMexA,$a$ = 30 dm^2^ is the surface of the monolayer, given by Equation (S16). $\gamma_{mF}$ and $\gamma_{mB}$ are the proportionality coefficients that relates the concentration of mMexA and ${mA}_{1}M$ to the observed fluorescence.

We plotted $I_{mF}+I_{mB}$ with time (Figure S6A). It remains constant over time (the dashed line gives the average value) indicating that there is no change in fluorescence emission when mMexA binds to an OprM and thus $\gamma_{mF}$ and $\gamma_{mB}$ are equal. This result proves that the intensities give a direct monitoring of the concentrations of the corresponding species and confirms the hypothesis made in Supplementary Information, section 1.


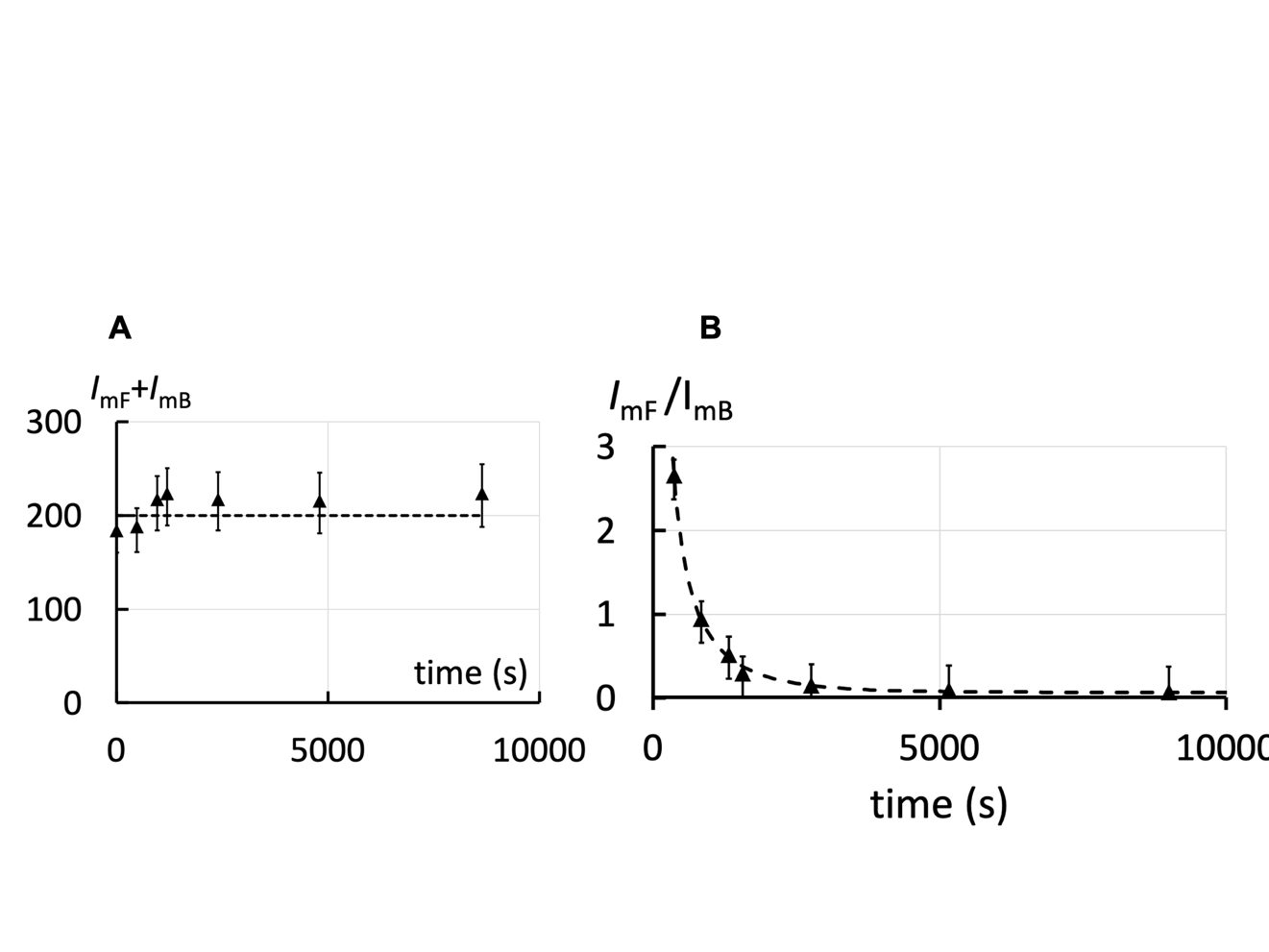


Fig. S6. (A) Evolution of the sum $\boldsymbol{I}_{\boldsymbol{mF}}\boldsymbol{+}\boldsymbol{I}_{\boldsymbol{mB}}$ with time. This quantity is almost constant (the dashed line gives the average value). The proportionality coefficients between intensities and concentrations $\boldsymbol{\gamma}_{\boldsymbol{mF}}$ for the free protein mMexA and $\boldsymbol{\gamma}_{\boldsymbol{mB}}$ for the bounded protein $\boldsymbol{m}\boldsymbol{A}_{\boldsymbol{1}}\boldsymbol{M}$ are equal (Equation (S37)). (B) Evolution of the ratio $\boldsymbol{I}_{\boldsymbol{mF}}\boldsymbol{/}\boldsymbol{I}_{\boldsymbol{mB}}$ with time. A fit of this quantity (Equation (S44)) allows to deduce the $\boldsymbol{k}_{\boldsymbol{on,2.5}\boldsymbol{d}}$ and $\boldsymbol{k}_{\boldsymbol{off}}$ of the reaction.

Furthermore, the dynamics of the complex formation is described by the kinetic equation:

$$\frac{d}{dt}\left[ mA_{1}M \right]=k_{on,2.5d}\left\{ mMexA \right\}\left[ OprM \right]-k_{off}\left[ mA_{1}M \right] (S38)$$

where $k_{on,2.5d}$ and $k_{off}$ are the association and dissociation rates.

The quantity of mMexA protein is conserved during the experiment. We thus can write a conservation equation:

$$V_{eff}\left\{ mMexA \right\}+a\left[ {mA}_{1}M \right]=n_{{mMexA}_{0}} (S39)$$

As the OprM is in large excess, [OprM] is supposed to be constant (= 2.10^-12^ mol.dm^-2^). Solving the system composed of Equation (S38) and Equation (S39), we obtain:

$$\frac{d}{dt}\left[ {mA}_{1}M \right]+ \frac{1}{\tau}\left[ mA_{1}M \right]= k_{on,2.5d}\left[ OprM \right]\frac{n_{{mMexA}_{0}}}{V_{eff}} (S40)$$

where:

$$\frac{1}{\tau}=k_{off}+k_{on,2.5d}\frac{a}{V_{eff}}\left[ OprM \right] (S41)$$

The boundary condition is that at the beginning of the experiment, there is not any mMexA that is bounded to OprM: $\left[ {mA}_{1}M \right]_{0}$ = 0. The deduced evolutions of the concentrations of mMexA and its complex with OprM are:

$$\left\{ \begin{aligned} \left[ {mA}_{1}M \right]= k_{on,2.5d}\frac{n_{{mMexA}_{0}}}{V_{eff}}\left[ OprM \right]\tau(1-e^{-\frac{t}{\tau}}) \\ \left\{ mMexA \right\}= \frac{n_{{mMexA}_{0}}}{V_{eff}}(1-k_{on,2.5d}\frac{a}{V_{eff}}\left[ OprM \right]\tau\left( 1-e^{-\frac{t}{\tau}} \right)) \end{aligned} \right. (S42)$$

We can then express the {mMexA}/[${mA}_{1}M$] ratio :

$$\frac{\left\{ mMexA \right\}}{\left[ {mA}_{1}M \right]}=\frac{1}{k_{on,2.5d}\left[ OprM \right]\tau(1-e^{-\frac{t}{\tau}})}-\frac{a}{V_{eff}} (S43)$$

From Equation (S37) and (S43) we deduce:

$$\frac{I_{mF}}{I_{mB}}=\frac{V_{eff}}{k_{on,2.5d}a\left[ OprM \right]\tau(1-e^{-\frac{t}{\tau}})}-1 (S44)$$

Like for the streptavidin-biotin interaction, $V_{eff}$ can be easily deduced from the hydrodynamic radius $r_{H}$of the soluble protein which can be obtained from the diffusion of mMexA in solution after the use of the Stokes-Einstein equation, or it can be estimated by the Perrin’s formulas^3^. Considering mMexA to be an ellipsoid, with a 1.5 nm radius and an 8.5 nm height^4^, we obtain a realistic value $r_{H}$ ≈ 2.1 nm. We deduce $V_{eff}$ = 8.89 µL.

Two unknown parameters are left in Equation (S44): $k_{on,2.5d}$ and $\tau$. Since our experiment gave the evolution of $I_{mF}$ and $I_{mB}$ overtime, we fit the ratio in Equation (S49) to deduce $k_{on,2.5d}$ and $\tau$ (Figure S6B). Actually, $\tau$ is the characteristic time of $I_{mF}$ and $I_{mB}$ variations which are fitted in Figure 3A and 3B. It is equal to 1061 ±136 s. We then deduce $k_{off}$ from Equation (S41).

**S8. Diffusion of native MexA with or without OprM in solution**


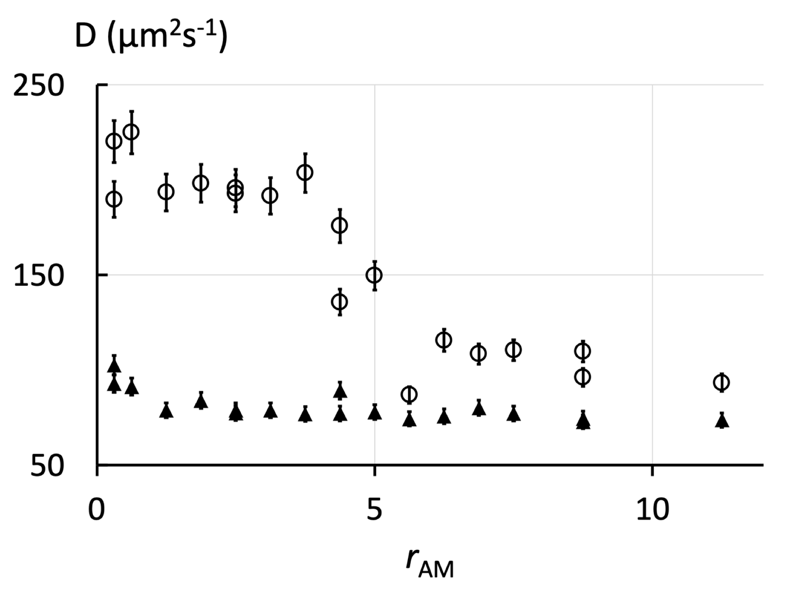


Fig. S7. Variation of diffusion coefficients $\mathbf{D}$ with $\boldsymbol{r}_{\boldsymbol{AM}}$ of FITC-labeled MexA alone in solution (⭘) or in the presence of OprM (▲) at a concentration of 1.6 μM after 24 hours of incubation. The lower $\mathbf{D}$ values at low MexA concentrations show that MexA and OprM gather, either interacting with each other or gathering within the same detergent micelles. The decrease in the diffusion coefficients values of MexA alone with $\boldsymbol{r}_{\boldsymbol{AM}}$ is attributed to dimerization or limited aggregation of proteins.

References

1. Kurtisovski, E., Taulier, N., Ober, R., Waks, M. & Urbach, W. Molecular Origin of Model Membrane Bending Rigidity. *Phys. Rev. Lett.* **98**, 1–4 (2007).

2. Adrien, V. *et al.* Characterization of a Biomimetic Mesophase Composed of Nonionic Surfactants and an Aqueous Solvent. *Langmuir* **32**, 10268–10275 (2016).

3. Perrin, F. Mouvement brownien d’un ellipsoide - I. Dispersion diélectrique pour des molécules ellipsoidales. *J. Phys. Radium* **5**, 497–511 (1934).

4. Akama, H. *et al.* Crystal structure of the drug discharge outer membrane protein, OprM, of Pseudomonas aeruginosa: dual modes of membrane anchoring and occluded cavity end. *J. Biol. Chem.* **279**, 52816–52819 (2004).
